# The gut microbial metabolite formate exacerbates colorectal cancer progression

**DOI:** 10.1038/s42255-022-00558-0

**Published:** 2022-04-18

**Authors:** Dominik Ternes, Mina Tsenkova, Vitaly Igorevich Pozdeev, Marianne Meyers, Eric Koncina, Sura Atatri, Martine Schmitz, Jessica Karta, Maryse Schmoetten, Almut Heinken, Fabien Rodriguez, Catherine Delbrouck, Anthoula Gaigneaux, Aurelien Ginolhac, Tam Thuy Dan Nguyen, Lea Grandmougin, Audrey Frachet-Bour, Camille Martin-Gallausiaux, Maria Pacheco, Lorie Neuberger-Castillo, Paulo Miranda, Nikolaus Zuegel, Jean-Yves Ferrand, Manon Gantenbein, Thomas Sauter, Daniel Joseph Slade, Ines Thiele, Johannes Meiser, Serge Haan, Paul Wilmes, Elisabeth Letellier

**Affiliations:** 1 Molecular Disease Mechanisms Group, Department of Life Sciences and Medicine, Faculty of Science, Technology and Medicine, University of Luxembourg, Belvaux, Luxembourg; 2 School of Medicine, National University of Ireland, Galway, Ireland; 3 Ryan Institute, National University of Galway, Galway, Ireland; 4 Cancer Metabolism Group, Department of Cancer Research, Luxembourg Institute of Health, Luxembourg, Luxembourg; 5 Department of Biochemistry, Virginia Polytechnic Institute and State University, Blacksburg, VA USA; 6 Systems Ecology Group, Luxembourg Centre for Systems Biomedicine, University of Luxembourg, Esch-sur-Alzette, Luxembourg; 7 Integrated BioBank of Luxembourg, Dudelange, Luxembourg; 8 National Center of Pathology, Laboratoire National de Santé, Dudelange, Luxembourg; 9 Department of Surgery, Centre Hospitalier Emile Mayrisch, Esch-sur-Alzette, Luxembourg; 10 Clinical and Epidemiological Investigation Center, Department of Population Health, Luxembourg Institute of Health, Luxembourg, Luxembourg; 11 Discipline of Microbiology, School of Natural Sciences, National University of Ireland, Galway, Ireland; 12 APC Microbiome, Cork, Ireland

**Keywords:** Cancer metabolism, Bacterial host response, Colorectal cancer, Metabolism

## Abstract

The gut microbiome is a key player in the immunomodulatory and protumorigenic microenvironment during colorectal cancer (CRC), as different gut-derived bacteria can induce tumour growth. However, the crosstalk between the gut microbiome and the host in relation to tumour cell metabolism remains largely unexplored. Here we show that formate, a metabolite produced by the CRC-associated bacterium *Fusobacterium nucleatum*, promotes CRC development. We describe molecular signatures linking CRC phenotypes with *Fusobacterium* abundance. Cocultures of *F. nucleatum* with patient-derived CRC cells display protumorigenic effects, along with a metabolic shift towards increased formate secretion and cancer glutamine metabolism. We further show that microbiome-derived formate drives CRC tumour invasion by triggering AhR signalling, while increasing cancer stemness. Finally, *F. nucleatum* or formate treatment in mice leads to increased tumour incidence or size, and Th17 cell expansion, which can favour proinflammatory profiles. Moving beyond observational studies, we identify formate as a gut-derived oncometabolite that is relevant for CRC progression.

## Abstract (web summary)

Different gut bacteria have been shown to promote colorectal cancer (CRC) progression. The authors identify formate as an oncometabolite derived from *Fusobacterium nucleatum*, which promotes CRC formation by increasing cancer stemness.

## Main

Colorectal cancer (CRC) is one of the most frequently diagnosed types of cancer worldwide, ranking third in terms of tumour incidence and second in cancer mortality^1^. The gut microbiome has been associated with molecular mechanisms underlying CRC initiation, progression and treatment. Isolated gut microbes have been shown to induce genetic and microenvironmental changes, ultimately supporting cancerous cell growth. In the past decades, extensive taxonomic and functional profiling of the CRC-associated microbiome has emerged. Along with ^pks+^*Escherichia coli*, enterotoxigenic *Bacteroides fragilis* and *Streptococcus* *gallolyticus*, *F. nucleatum* (*Fn*) isolates were identified as a major protumorigenic bacterium. However, in CRC microbiome research, there is an urgent need to move beyond descriptive studies.

Sophisticated study models, such as organoids and gut-on-chip models, can help advance microbiome research in a mechanistic direction. The gut organoid model was recently used to reveal a causative role of a genotoxic ^pks+^*E. coli* mutational signature in CRC^2^. In vitro modular perfusion bioreactor systems, such as HuMiX, have been used to study host metabolic, transcriptional and immune responses to microbes in CRC^3,4^. Moreover, genome-scale metabolic models based on human gut metagenomics data show how microbes can modulate the human metabolism in health and disease, ultimately predicting drug- or dietary-based therapies for CRC^5,6^. Finally, mouse models remain the golden standard in CRC microbiome research, allowing for the study of tumours, their microenvironment and their immune landscape^7^.

The integral results of published in vitro, in vivo and in silico models strongly support a scenario whereby *Fn* colonizes tumours and promotes CRC growth. Several virulence factors are involved in this process, such as Fap2, FadA, RadD and FomA. Fap2 contributes to tumour colonization and bacterial invasion into Gal-GalNAc expressing epithelial cells^8^. Furthermore, Fap2 binding to host cells increases IL8 and CXCL1 production, driving cancer cell migration^9^ and tumour immune evasion^10^. FadA mediates host-cell binding and invasion and promotes cancer cell proliferation^11^. RadD can cause host-cell death and bridge tumour colonization for other pathogens in biofilms^12^. FomA stimulates TLR2-dependent NF-κB signalling in gut epithelial cells^13^. Elaborated secretome analyses on *Fn* are providing further insights on putative and known virulence proteins^14^, yet virulence expression is tightly controlled and only subordinate to pathogen metabolism^15^. In particular, microbiome-derived oncometabolites, such as l-2-hydroxyglutarate, succinate, fumarate, d-2-hydroxyglutarate or lactate can accumulate in cancers, thereby fuelling malignancy^7^. Moreover, oncometabolites can hijack metastatic signalling via gene expression regulation^16^. In this context, very little is known about the fusobacterial metabolic contribution to a malignant state.

*Fusobacteria* have previously been shown to migrate to the metastatic site^17^, potentially shuttled by disseminated cells via the hematogenous route^9^, demonstrating *Fusobacterium*’s persistence throughout the metastatic process. It has been suggested that metastatic colonization (that is, outgrowth at a secondary site) is restricted to cancer cells with tumour-initiating properties (cancer stem cells, CSCs)^18^. CSCs are defined by key functional features including their ability to self-renew and replenish tumour heterogeneity, thereby driving tumour initiation and progression^19^. *Fn* was shown to induce CSC characteristics in CRC cells in vitro^20^, which can be associated with late-stage *Fn* positive cases^21^. Nevertheless, the mechanisms behind *Fn*-associated cancer stemness and invasion remain unclear.

In this study, we aimed to integrate in vitro, in vivo and in silico approaches to gain an ecosystem-level mechanistic understanding of *Fn*’s metabolic role in CRC pathogenesis. We propose that *Fn* not only directly interacts with and contributes to CRC cell malignancy, but that its microbe–host metabolic crosstalk is involved in the progression of the disease. More specifically, we identified the small-molecule formate as an oncometabolite that contributes to CRC stemness, invasion and metastasis.

## Results

### *Fn* is elevated in patients with CRC and associated with consensus molecular subtype 3 (CMS3)

**Fig. 1:**
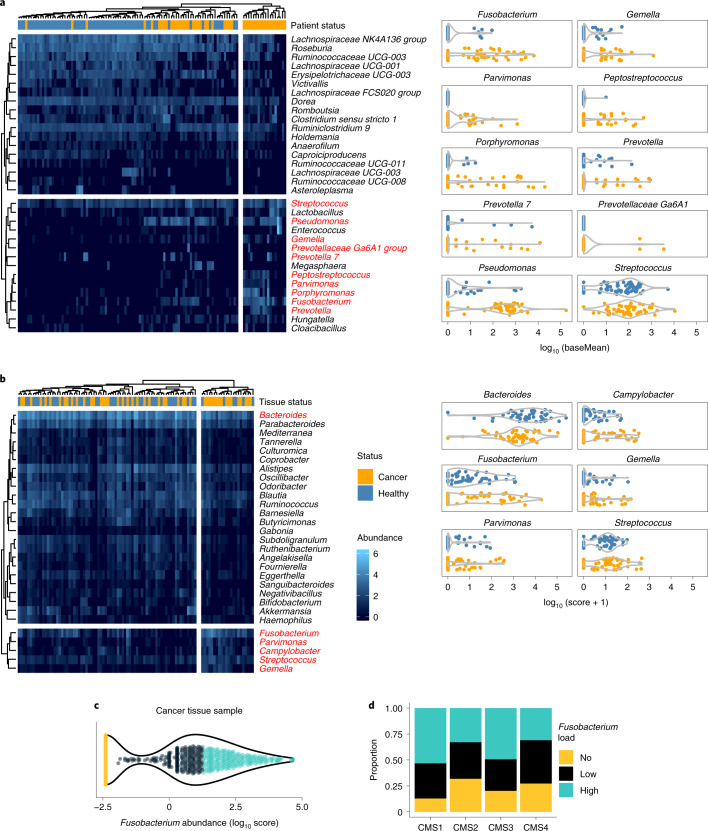
*Fusobacterium* levels are elevated in stool and tissue from patients with CRC and are associated with CMS1 and metabolic-driven CMS3. **a**, Differential abundance of bacterial genera in CRC stool samples. Clustered heatmap (Pearson correlation) shows the significant differentially abundant bacterial genera in an in-house cohort of samples from patients with CRC (*n* = 52 independent biological samples) in comparison with healthy donor (*n* = 63 independent biological samples) (*P* < 0.05; negative binomial Wald Benjamini–Hochberg testing) as identified by 16S rRNA gene sequencing. Top CRC-enriched bacteria^7^ are marked in red and separately plotted as abundance (baseMean) of a bacterium per patient (right). Heatmap intensities (blue colour scale) represent log_10_ base mean values. **b**, Differential abundance of bacterial genera in CRC-tissue samples, following a PathSeq analysis on whole-exome sequencing (WXS) data (TCGA). Clustered heatmap (Pearson correlation) showing the significant differentially abundant bacterial genera in tumour tissue samples from patients with CRC (*n* = 50) in comparison with adjacent healthy mucosal samples (*n* = 50, *P* < 0.05; negative binomial Wald Benjamini–Hochberg testing). Top CRC-enriched bacteria are marked in red and separately plotted as abundance (score) of a bacterium per sample (right). Heatmap intensities (blue colour scale) represent log_10_ scores. **c**, *Fusobacterium* abundance in tissue samples from patients with CRC (*n* = 595 independent biological samples, TCGA). WXS data were analysed for the logarithmic score abundance of *Fusobacterium* across all samples via quantile-based classification (colour code). **d**, *Fusobacterium* abundance across CMS subtypes. The cohort (**c**) was subjected to gene expression analysis and CMS classification of matching RNA-seq^67^. Coloured, segregated bars show the proportion of patients with fusobacterial loads per CMS. Significant differences were observed for CMS1 versus CMS2 (*P* = 0.003103733) or CMS4 (*P* = 0.006062911) and CMS3 versus CMS2 (*P* = 0.038151361) or CMS4 (*P* = 0.035902419) Chi-squared tests. *n*^CMS1^ = 62, *n*^CMS2^ = 207, *n*^CMS3^ = 69, *n*^CMS4^ = 139 biologically independent samples. Source data

To investigate the CRC microbiome in an in-house cohort (Supplementary Table 1), we analysed the bacterial compositions of stool samples from patients with CRC (*n* = 63) and compared them with the stool microbiomes of a healthy cohort (*n* = 52) using 16S ribosomal RNA gene sequencing. The differential abundance analysis (Fig. 1a) identified bacterial genera that were significantly depleted in CRC donor stools, such as the potential probiotics Ruminococcaceae and Lachnospiraceae, and genera that were significantly enriched, such as the well-described *Fusobacterium*, *Porphyromonas*, *Pseudomonas*, *Streptococcus* and *Gemella* (marked in red).

**Extended Data Fig. 1:**
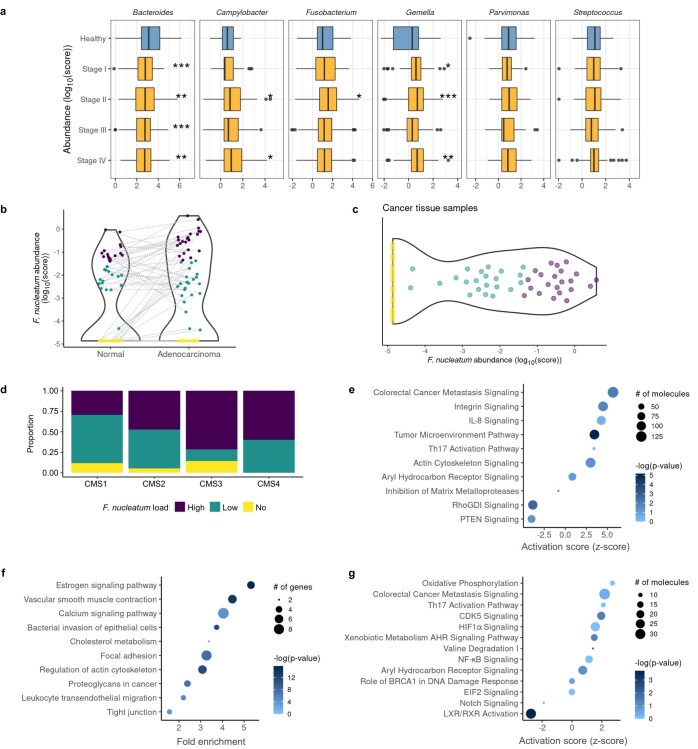
Microbiome composition in CRC patients and pathways identified in *Fusobacterium*^high^ patients. **a**, Abundance of top CRC-enriched and differentially abundant bacteria in tissue per stage (TCGA). Boxplots shows medians with 1^st^ and 3^rd^ quantiles. The whiskers from the hinges to the smallest/largest values represent 1.5*inter-quartile range (IQR). n=108 healthy, n=102 Stage I, n= 209 Stage II, n=163 Stage III and n=86 Stage IV biologically independent donor samples, p=0.000705, p=0.00101, p=0.0000154 and p=0.00153 for *Bacteroides* in Stages I-IV vs. Healthy respectively, p=0.0112 and p=0.0402 for *Campylobacter* in Stages II and IV vs. Healthy respectively, p=0.0223 for Fusobacterium in Stage II vs Healthy, p=0.0293, p=0.000608 and p=0.00267 for Gemella in Stages I, II and IV vs. Healthy respectively, pairwise t test. **b**, *Fn* abundance in the EGA cohort. PathSeq-analysis for *Fn* abundance in matching normal vs. adenocarcinoma tissue on RNA-seq data, p=0.00165, paired two-tailed t test. **c**, *Fn* tissue abundance distribution in CRC patients of the EGA cohort. *Fn* was detected via RNA-seq and analyzed for the logarithmic score bacterial distribution (n=69). Quantile-based classification (color code) was applied for the target bacterium (quantcut function from the *gtools* R package). **d**, Correlation of *Fn* abundance with consensus molecular subtypes in CRC. The cohort in **c** was subjected to gene expression analysis and further classified via the *CMScaller* R package into CMS as described^67^. Colored, segregated bars show the proportion of patients with differing fusobacterial loads per CMS. Chi-squared tests were performed for comparing the fusobacterial across all CMS. No significant differences were observed. n^CMS1^=17, n^CMS2^=19, n^CMS3^=7, n^CMS4^=5. **e**, IPA analysis of *Fusobacterium*^high vs. no^ differential gene expression analysis of the TCGA dataset. Plot shows z-scores, p-values, and the number of molecules per pathway. Selected significant pathways are shown (-log(p-value)>1.3). **f**, KEGG-based GSEA of *Fusobacterium*^high vs. no^ differential gene expression analysis of the TCGA dataset (pathfindR R package). Plot shows fold enrichment, p-values, and the number of genes per pathway. All significant pathways are shown (p<0.05). **g**, IPA analysis of *Fusobacterium*^high vs. no^ differential gene expression analysis of the EGA dataset. Plot shows z-scores, p-values, and the number of enriched molecules per pathway. Selected significant pathways are shown (-log(p-value)>1.3). *p<0.05, **p<0.01, ***p<0.001. Source data

We used PathSeq, a valuable tool for the analysis of metagenomic data^22^, to identify CRC-tissue associated bacterial genera and species in datasets from The Cancer Genome Atlas (TCGA) (*n* = 595) and the European Genome-Phenome Archive (EGA) (*n* = 69). The analysis of the tissue microbiome identified bacteria that were associated with a cancerous tissue state (Fig. 1b). Among these bacteria were *Fusobacterium*, *Parvimonas*, *Gemella* and *Streptococcus* (marked in red). The *Bacteroides* genus was depleted in tumour tissue across all stages of the disease and elevated abundances of *Fusobacterium* were observed in stage II CRC, *Campylobacter* in stages II and IV, and *Gemella* in stages I, II and IV (Extended Data Fig. 1a). Of these bacteria, *Fn* was also enriched in tumour tissues in the EGA cohort, when compared with matching adjacent tissue (Extended Data Fig. 1b, *P* < 0.02), which has also been previously observed in independent datasets^23,24^.

TCGA and the EGA databases contain valuable gene expression data from patient tissue, allowing us to link patient transcriptomic and microbial profiles (metadata for these datasets can be found in Supplementary Tables 2 and 3). We were particularly interested in associating higher levels of *Fusobacteria* in patients to the CMS classification of CRC. This classification uses the transcriptomic profile of tumours to group them into CMS1 (hypermutated and immunogenic), CMS2 (characterized by the activation of the WNT and MYC pathways), CMS3 (metabolically deregulated) and CMS4 (strong stromal infiltration associated to lower relapse-free and overall survival expectancies)^25^. To this end, we classified patients into no (score, 0), low (score, ≤*t*, where *t* is the median value of positive scores) and high (score, >*t*) fusobacterial abundance (Fig. 1c). The results show that CMS1 and CMS3 patients have a similarly high proportion of *Fusobacterium* (*χ*^2^(2, *n* = 595) = 1.3, *P* > 0.05), which was significantly different from the proportions observed in CMS2 and CMS4 patients (*P* < 0.05) (Fig. 1d). This observation is also in agreement with very recent work from Salvucci and collaborators^26^. In addition, CMS3 tended to harbour high proportions of *Fusobacterium*^high^ cases in the EGA dataset (Extended Data Fig. 1c,d). We therefore suggest that *Fusobacterium* not only plays a role in CMS1 (ref. ^24^), but also in CMS3, which is enriched in metabolic pathways including glutamine, fatty acid and lysophospholipid metabolism^27^. Next, we performed differential gene expression analyses comparing *Fusobacterium*^high^ to *Fusobacterium*^no^ cases. Our pathway enrichment analyses showed that inflammatory-related signalling pathways, such as IL-8 signalling or Th17 activation, were upregulated (Extended Data Fig. 1e). In addition, pathways related to cellular organization, movement and invasion, as well as metabolic pathways involving cholesterol and proteoglycan metabolism, were affected (Extended Data Fig. 1e,f). Notably, aryl hydrocarbon receptor (AhR) signalling was found activated in both the TCGA and the EGA datasets when *Fusobacteria* were abundant (Extended Data Fig. 1e,g).

### *Fn* induces protumorigenic signalling and formate secretion

**Fig. 2:**
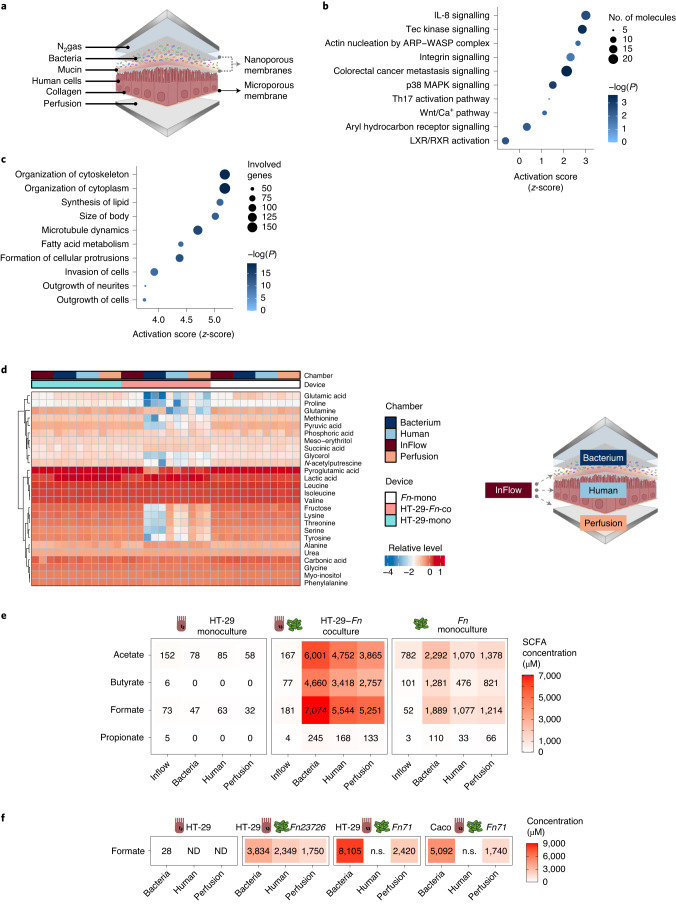
Gut-on-chip cocultures (HuMiX) of *Fn* with patient-derived primary CRC cells reveal protumorigenic and pro-invasive effects along with an altered metabolic profile. **a**, Schematic representation of the HuMiX setup. **b**, IPA canonical pathway analysis of HuMiX HT-29 co- versus monoculture differential gene expression analysis. Plot shows *z*-scores, -log(*P)* values and the number of molecules per pathway. Selected significant pathways are shown (*P* < 0.05), Fisher’s Exact Test. **c**, IPA Diseases or Functions analysis of HuMiX HT-29 co- versus monoculture differential gene expression analysis. Plot shows *z*-scores, *P* values, and the number of genes per pathway. Top ten significant pathways are shown (*P* < 0.05): Fisher’s exact test. **d**, Exometabolite profile of HuMiX cocultures. Untargeted GC–MS metabolite detection in chamber supernatants was used to measure relative, normalized metabolite levels. Heatmap shows relative log intensities of extracellular metabolites (rows) accross *n* = three independent experiments, each with one device per condition (main columns) composed of four chambers (subcolumns). **e**, SCFA levels in the HuMiX after HT-29-*Fn* coculture. Heatmap shows mean concentrations of extracellular SCFAs (rows) from *n* = 3 independent experiments, each with one HuMiX device per condition, composed of four chambers (columns). **f**, Levels of formate in the HuMiX chambers using different *Fn* isolates (*Fn* 23726 and one clinical isolate *Fn* ssp. *animalis* 7_1) in coculture with HT-29 or Caco-2 cells (*n* = 1 experiment with one HuMiX device per condition). ND, not detected and n.s., not sampled. Source data

**Extended Data Fig. 2:**
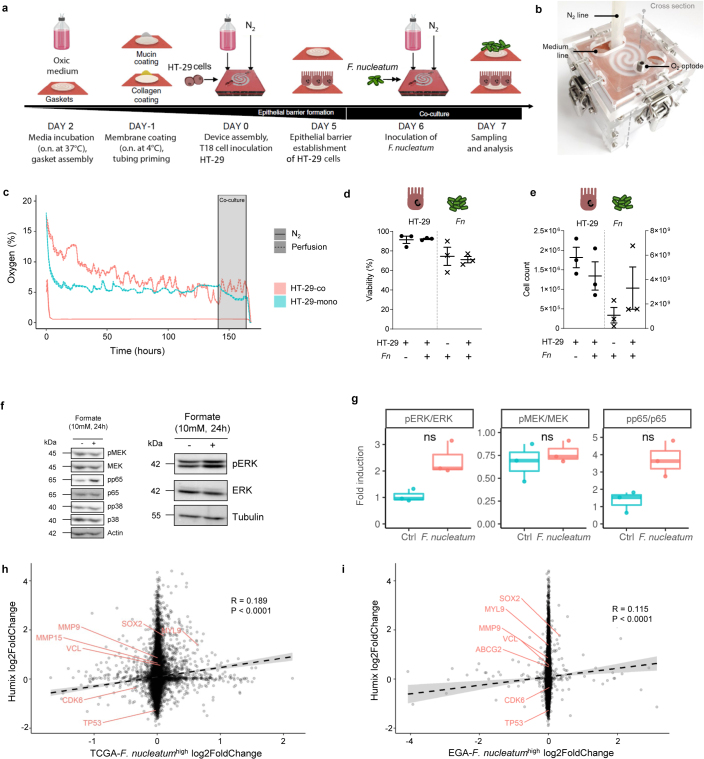
Microbiome cross-talk studies in HuMiX. **a**, Experiment layout of HuMiX experiments. Devices assembly and medium priming for 1 day; epithelial cell inoculation and monolayer formation for 6 days; bacterial-human coculture for 24h. Control devices with either bacterial or human cell mono-cultures were run in parallel. At the endpoint, devices were opened for chamber metabolite sampling and cell harvesting. **b**, Clamped HuMiX device, showing the HuMiX device (spiral), the N_2_ and medium lines as well as free optode pockets (cf. Figure 2A). **c**, Oxygen levels (%) as monitored in the perfusion and N_2_ gas microchambers of HuMiX HT-29 mono-/ and cocultures. The grey box marks the the co-culture time. **d**, *Fn* and HT-29 cell viability in HuMiX mono-/ and co-cultures. Human cell viability was assessed by TrypanBlue staining and FACS-based L/D NearIR staining. Bacterial cell viability was assessed by BacLight L/D staining under a microscope. Three representative images were taken and cell viability was assessed with ImageJ. (**c**), (**d**) and (**e**) show data from n=three biologically independent experiments. **e**, Cell counts of human cells and bacteria under co-culture. Cell counts of HT-29 cells were taken after TrypanBlue staining. Bacteria cell counts were assessed using CASY cell counter with the 45µm capillary. **f**, Western Blot detection of MAPK signaling, as well as NF-κB signaling on HuMiX HT-29 cell lysates. phosphorylation of MEK at Ser217/221, p65 at Ser536, pERK at thr 202/tyr204, and p38 at thr180/tyr182 under HT-29 monoculture (-) and HT-29-*Fn* co-culture (+) conditions in HT-29 cells. Data shows one representative of three biologically independent experiments. **g**, Quantified ratios of phosphorylated protein per total target protein levels from (**f**), n=three biologically independent experiments. All boxplots show medians with 1^st^ and 3^rd^ quantiles. The whiskers from the hinges to the smallest/largest values represent 1.5*inter-quartile range (IQR). ns=not significant; two-sided paired t-tests. **h-i**, Correlation of global transcriptomic profile of HuMiX co-cultures with *Fusobacterium*^high vs. no^ patient’s gene expression profile of the TCGA (**h**) and EGA (**i**) datasets. Interesting gene targets are marked in red. Two-tailed Spearman’s rho correlation testing was used to assess significance between the two data sets. Source data

To specifically address how *Fn* influences tumour cell metabolism, we used the modular, perfusion bioreactor system HuMiX. The model previously helped us to study metabolic, transcriptional, and immune responses of CRC cells in coculture with the probiotic *Lactobacilli*^4^. HuMiX represents a unique system to study host-microbiome metabolic crosstalk where the exchanged metabolites can be independently analysed in each chamber. To test the effects of host–*Fusobacterium* crosstalk, we cocultured the patient-derived, CRC cell line HT-29 with *Fn* ATCC 25586 in HuMiX (Fig. 2a and Extended Data Fig. 2a,b). HT-29 cells were obtained from ATCC and were derived from a 44 year old female patient with grade II adenocarcinoma and selected on the basis of the stage II enrichment of *Fn* in patients with CRC (Extended Data Fig. 1a). The HuMiX system allowed us to study the effects of *Fn* on cancer cells at near-physiological oxygen levels (Extended Data Fig. 2c) and under a constant supply of fresh medium to the basal cell surface. The 24-hour coculture had no effect on cell viability, nor on *Fn* and HT-29 proliferation (Extended Data Fig. 2d,e). The transcriptional response of HT-29 cells to *Fn* exposure showed effects on Wnt, IL8 and mitogen-activated protein kinase (MAPK) downstream pathway activation (Fig. 2b), traits that have been linked with *Fusobacteria* in CRC in the past^11,28^. Further activated pathways included Th17 activation, AhR receptor signalling and CRC metastasis signalling. Moreover, when investigating potential functional effects, the ingenuity pathway analysis (IPA) predicted high activation scores for cancer cell morphology and invasion (Fig. 2c). To confirm this functional regulation, we looked at the activation of the MAPK signalling pathway, which is important for multiple biological processes (for example, cell proliferation, differentiation, migration and invasion). As typical responders to extracellular stimulation, the phosphorylation of extracellular signal-regulated kinase (ERK) and slight activation of MEK and p38 were observed on the molecular exchange between *Fn* and HT-29 (Extended Data Fig. 2f,g). In addition, phosphorylation of the p65 (NF-κB subunit) tended to be increased due to *Fusobacterium* (Extended Data Fig. 2f,g).

**Extended Data Fig. 3:**
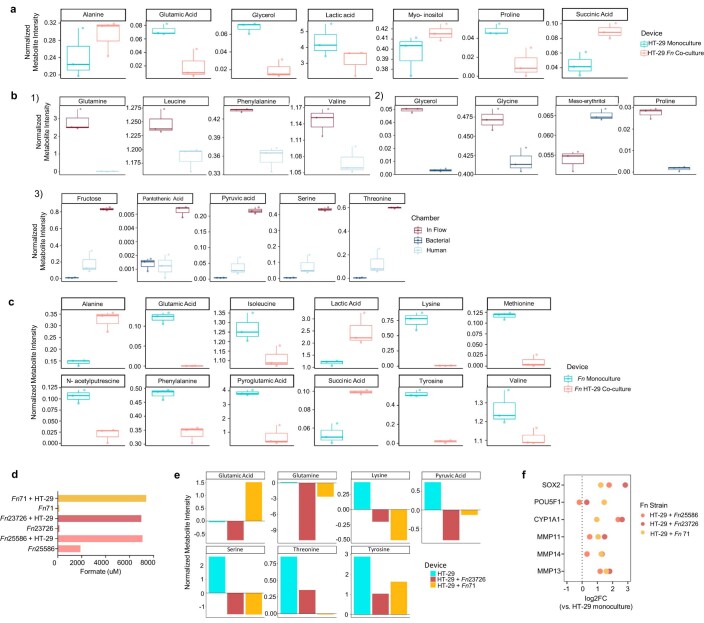
Metabolome composition in HuMiX microbiome-host crosstalk. **a**, Significant differentially abundant metabolites in HT-29 mono-/ vs. HT-29-co-cultures, human cell microchamber supernatants. **b**, Significant differentially abundant metabolites in **(1)** HT-29-co-cultures comparing inflow metabolite levels with human chamber metabolite levels, **(2)** *Fn*-co-cultures, comparing inflow metabolite levels with bacterial microchamber supernatants. **(3)**, HT-29-co-cultures and *Fn*-co-cultures, comparing inflow metabolite levels with human and bacterial microchamber supernatants. These were ambiguous metabolites that could not be assigned to any of the two cell types as being produced or secreted. **c**, Significant differentially abundant metabolites in *Fn* mono-/ vs. *Fn*-co-cultures, bacteria microchamber supernatants. **a**, **b** and **c** show data from n=three biologically independent experiments. All boxplots (in **a**, **b** and **c**) show normalized median metabolite levels (log intensities). Boxplots show medians with 1^st^ and 3^rd^ quantiles. The whiskers from the hinges to the smallest/largest values represent 1.5*inter-quartile range (IQR). p<0.05 for **a**, **b**, and **c**; unpaired, two-sided t-test. **d**, Levels of formate in different *Fn* isolates (*Fusobacterium nucleatum* 23726 and *Fusobacterium nucleatum* ssp. animalis 7_1, clinical isolate, one chamber per isolate) in monoculture and co-cultures with T 18 cells in HuMiX. **e**, Levels of metabolites in HuMiX in HT-29 cells alone or in co-culture with different Fn isolates (*Fusobacterium nucleatum* 23726 and *Fusobacterium nucleatum* ssp. animalis 7_1, clinical isolate, one chamber per isolate). **f**, Genes related to AhR signaling and cancer stemness in HT-29 cells after co-culture with different *Fn* isolates in HuMiX. Source data

The global transcriptional changes observed in the HuMiX experiments, including the activation of Th17 and AhR receptor signalling, but also the induction of cancer stemness genes (for example *SOX2*), correlated with those observed in tissues from *Fusobacterium*^high^ patients (Extended Data Fig. 2h,i, see also Fig. 2b in comparison with Extended Data Fig. 1e,g). To address the soluble factors underlying the observed effects, we performed untargeted and targeted metabolite analyses. The results of the untargeted analysis showed that HT-29 cell culture chambers contained lower glutamic acid and glutamine levels, suggesting a boosted consumption (Fig. 2d and Extended Data Fig. 3a,b). In addition, lower levels of lactic acid in the HT-29 chambers compared to the monoculture suggest a metabolic switch from glucose to glutamine as the main carbon source. Regarding fusobacterial metabolism, large metabolite level increases were observed for succinic acid, lactic acid and alanine (Extended Data Fig. 3c). A range of amino acids, such as isoleucine, lysine, methionine, phenylalanine, tyrosine and valine were reduced in the coculture bacterial chambers compared to the respective monoculture chambers and the inflow metabolite profile, suggesting fusobacterial use (Extended Data Fig. 3b,c). The results from the targeted gas chromatography–mass spectrometry (GC–MS) analyses on exometabolites from the HuMiX chambers indicated that upon coculture, *Fn* secreted almost four times more acetate, propionate, butyrate and formate; the latter being the predominant secretion product (Fig. 2e). To verify that the increase in formate and other metabolites is common to different *Fn* strains when interacting with different CRC cells, we performed further HuMiX-based experiments focused on the metabolic crosstalk using different *Fn* strain isolates (*Fn* 23726 and one clinical isolate *Fn* ssp. *animalis* 7_1). Analysis of the metabolic profile of the different strains in coculture with HT-29 or Caco-2 (human colon epithelial cell line) clearly showed that formate is a common *Fn*-derived metabolite that is secreted upon coculture between different human CRC-derived cells and *Fn* strains (Fig. 2f and Extended Data Fig. 3d). The clinical isolate showed the highest secretion of formate compared to ATCC 23726 and 25586 *Fn* strains, further supporting the relevance of formate secretion in human disease. We then focused on the most interesting metabolites in the untargeted metabolomics analysis identified in Fig. 2d and Extended Data Fig. 3. Most of the altered metabolites including glutamine, lysine, pyruvate, threonine and serine, were reduced in HT-29 cells after coculture with *Fn* (Fig. 2d), independently of the strain used (Extended Data Fig. 3e). In addition, we observed an increase in genes related to the AhR signalling pathway and cancer stemness in CRC cells upon coculture with the various *Fn* strains described above (Extended Data Fig. 3f). Altogether, these data indicate that our findings are generalizable among CRC cells and *Fn* strains.

### *Fn* induces a central carbon metabolism shift in tumour cells

**Fig. 3:**
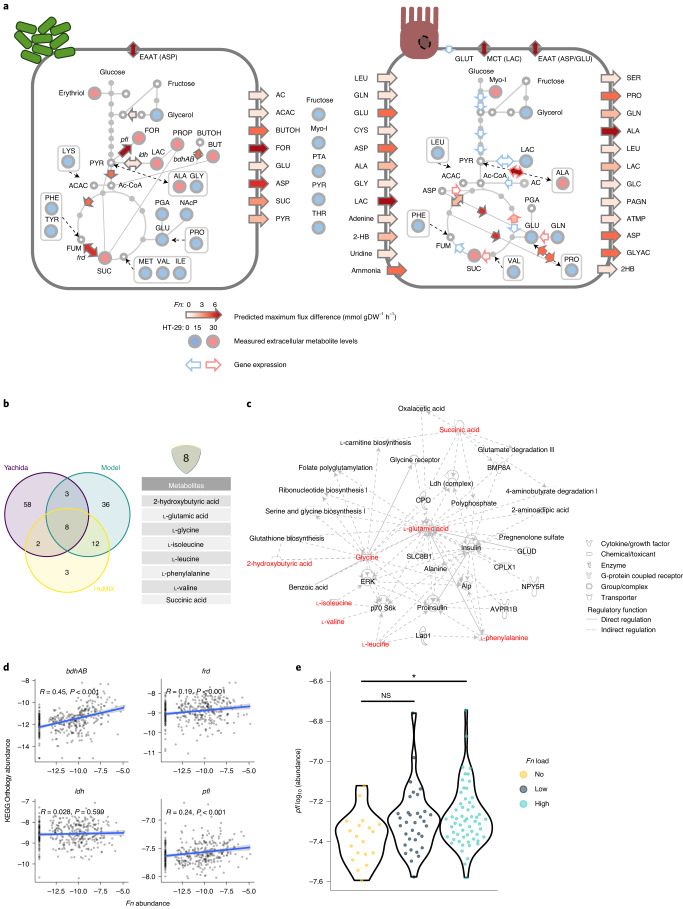
In silico modelling delineates enhanced formate metabolism of *Fn* in CRC context, which is also observed in *Fusobacterium*-high patients with CRC. **a**, Metabolic models of the central carbon metabolism of *Fn* (left) and HT-29 cells (right) in coculture versus monocultures. Biomass production was 0.20476 for *Fn* and 0.01 for HT-29. Flux constraints were set according to the metabolite secretion profiles of the HuMiX monoculture controls and pairwise interactions were calculated via FVA. Heatmap arrows show maximum flux changes (mmol dGW^−1^ h^−1^) of depicted reactions as predicted by FVA. Arrows at the cells’ boundaries represent intra-extracellular exchange reactions. Measured differentially abundant exometabolites in the respective HuMiX coculture chambers are shown as filled circles (blue, decreased; red, increased). Extracellular filled circles show unassigned metabolites. Coloured arrow borders show upregulated (red) or downregulated (blue) genes involved in the respective reaction fluxes, on the basis of RNA-seq data (refer to Fig. 2b). Dashed arrows show indirect connections between nodes. 2HB, 2-hydroxybutyrate; AC, acetate; ACAC, acetoacetate; ATMP, adenylthiomethyl-pentose; BUTOH, butanol; EAAT, excitatory amino acid transporters; GLYAC, glyceric acid; LAC, lactate; MCT, monocarboxylate transporters; NAcP, *n*-acetylputrescine; PAGN, phenylacetylglutamine; PGA, pyruglutamic acid; and *bdhAB*, butanol dehydrogenase. **b**, Core set of *Fn*-related metabolites. Venn diagram shows overlapping, differentially abundant metabolites in the Yachida dataset (purple), in HuMiX (yellow) and in the metabolic model (green). **c**, IPA network analysis of the gene-regulatory role of *Fn* core metabolites (red) in connection with host gene-regulatory nodes of stemness and invasion. **d**, KEGG orthology gene abundances of *Fn*-related genes identified by the model in patients with CRC with different fusobacterial load in stool samples. Two-tailed Spearman’s rho correlation testing was used. **e**, *pfl* in stool metagenomes of stage I/ II, *Fn*^high^ patients with CRC. *n*^Fn-no^ = 19, *n*^Fn-low^ = 34, *n*^Fn-high^ = 58 (NS, not significant, *P* = 0.0219 for *Fn*-no versus *Fn*-high; one-way analysis of variance (ANOVA) with Tukey’s honestly significant differences test). *P < 0.05. Source data

**Extended Data Fig. 4:**
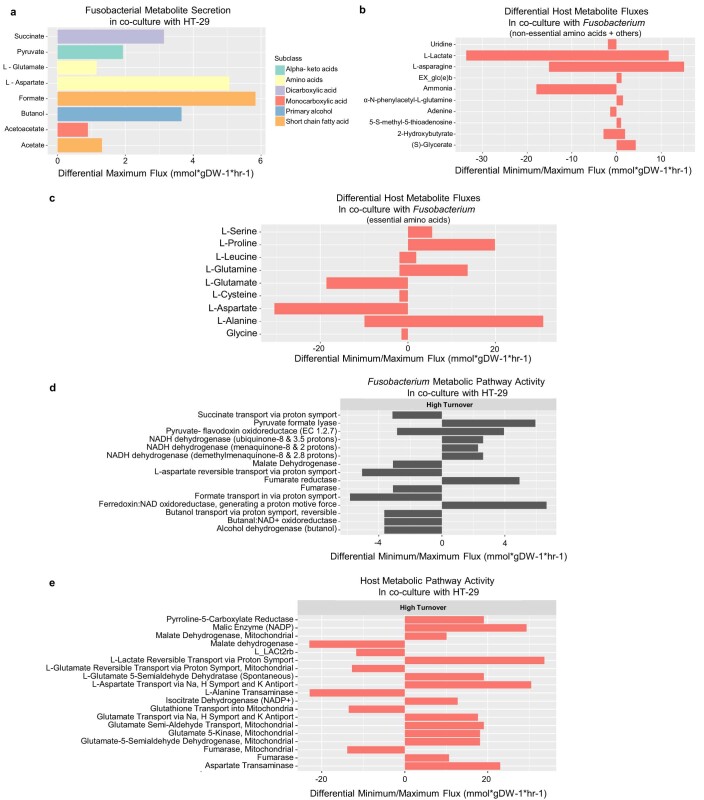
*In silico* metabolic modelling of Fn-host tumor cell interaction. **a**, Maximum flux changes for exchange reactions of *F. nucleatum* secreted metabolites. Calculated flux ranges from the FVA results of the *F. nucleatum* monoculture model were subtracted from FVA results of the HT-29-*Fn* co-culture model. No minimum flux changes were observed. **b**, Minimum and maximum flux changes for exchange reactions of HT-29 secreted/uptaken non-essential amino acids and other metabolites. Calculated flux ranges from the FVA results of the HT-29 mono-culture model were subtracted from flux ranges of the HT-29-*Fn* co-culture model. **c**, Minimum and maximum flux changes for exchange reactions secreted/uptaken essential amino acids in the HT-29 model. Calculated flux ranges from the FVA results of the HT-29 mono-culture model were subtracted from flux ranges of the HT-29-*Fn* co-culture model. **d**, Minimum and maximum flux changes for intracellular reactions of *F. nucleatum*. Calculated flux ranges from the FVA results of the *F. nucleatum* mono-culture model were subtracted from FVA results of the HT-29-*Fn* co-culture model. Cutoff for displayed reactions was -2 and 2 mmol*dGW^-1^*hr^-1^. **e**, Minimum and maximum flux changes for intracellular reactions of HT-29. Calculated flux ranges from the FVA results of the HT-29 mono-culture model were subtracted from flux ranges of the HT-29-*Fn* co-culture model. Cutoff for displayed reactions was -10 and 10 mmol*dGW^-1^*hr^-1^. Source data

To study the microbe–host crosstalk further, we used the *Fn* ATCC 25586 genome-scale reconstruction model from the AGORA resource^29^ as a basis for model curation and host–microbe pairwise interaction simulations. A Recon 2-based conditioned specific model of HT-29, grown in HuMiX, served as a host model^6^. The simulations of *Fn*–HT-29 cometabolism via flux variability analysis (FVA) predicted an increased production of formate when compared to the monoculture model. Notably, formate secretion to the extracellular space showed the biggest increase in maximum secretion flux, followed by aspartic acid and butanol (reflected in Fig. 3a, left cell, also Extended Data Fig. 4a). Acetate, succinate and glutamic acid were further metabolites, whose maximum secretion increased for *Fn*. These metabolites could serve as a carbon and nitrogen source, feeding into the cancer cells’ tricarboxylic acid (TCA) cycle. A predicted lower net production of lactate in HT-29 cells, together with a reduced gene expression of glycolysis enzymes and lower levels of lactate detected in the HT-29 HuMiX chamber (compared to its monoculture control), indicate a lower glycolytic activity of the CRC cells in coculture (reflected in Fig. 3a, right cell, also Extended Data Fig. 4b). In addition, the main glucose transporter GLUT1 was transcriptionally downregulated and the in silico model even allowed for an increased maximum glucose export flux, an effect potentially compensated by increased fluxes in glutamic acid and glutamine metabolism (Extended Data Fig. 4c). As indicated by RNA-sequencing (RNA-seq) and metabolomics data, TCA cycle enzyme activities were increased and pools of amino acids were reduced in HT-29 cell HuMiX chambers (Extended Data Fig. 4d,e). The in silico model supported this effect by predicting increased uptake rates for amino acids such as glutamine, glutamic acid, cysteine, aspartic acid, alanine and glycine, as the pools of these amino acids were depleted in HuMiX cocultures. Notably, *Fn* could provide an alanine source for HT-29 cells. Moreover, an additional pool of amino acids could be accessed by the HT-29 cells in coculture due to the intra and extracellular proteolytic activity of *Fusobacteria*^30^. Thus, serum protein breakdown could render additional nitrogen sources accessible to cancer cells.

To verify the patient relevance of our predicted and measured metabolite changes, we reanalysed metagenomic and metabolomic data from a Japanese cohort (Yachida; metadata for this cohort can be found in Supplementary Table 4)^31^. We compared how well the in silico model predicted the metabolic changes observed in the HuMiX experiments and in patients with high fusobacterial levels. A Venn diagram shows the overlapping differentially abundant metabolites from the three datasets (Fig. 3b). The constraint-based modelling approach covered 80% (20/25) of the differentially abundant metabolites in the HuMiX cocultures. An overlap of eight metabolites was shared among all three datasets: glutamic acid, 2-hydroxybutyrate, glycine, isoleucine, leucine, phenylalanine, succinic acid and valine. Of note, formate was left out in the analyte library of the Yachida dataset and is therefore not part of the list of the metabolites that are common between the three datasets. The IPA analysis for the downstream roles of these metabolites suggested a highly connected network of gene-regulatory nodes and metabolites involved in controlling stem cells and cellular invasion, as defined by IPA Disease and Function (Fig. 3c).

On the basis of the relative abundance of Kyoto Encyclopedia of Genes and Genomes (KEGG) orthology genes in patient stool metagenomes of the Yachida dataset, we checked for a correlation between previously identified bacterial enzymes (Extended Data Fig. 4d,e) and *Fn* abundance (Fig. 3d). Patients with higher *Fn* loads exhibited higher levels of butanol dehydrogenase (*bhdAB*), fumarate reductase (*frd*) and pyruvate-formate lyase (*pfl*) (*rho* > 1, *P* < 0.05). Lactate dehydrogenase (*ldh*) did not correlate with fusobacterial abundance. These results indicate that the genes for *Fn*-related metabolic interactions with tumour cells and especially formate production were increasingly present in patients with higher *Fn* levels. Accordingly, the KEGG orthology gene for microbial formate production *pfl* was significantly higher in stage I/II CRC patients who had high fusobacterial presence (Fig. 3e).

These results demonstrate that in coculture, *Fusobacterium* metabolism is altered towards the higher production of formate and the secretion of metabolites that potentially affect the function and regulation of malignant cells in CRC. At the same time, HT-29 cell central carbon metabolism relied more on TCA cycle linked metabolites. While model predictions such as increased maximum secretion flux for glucose or glutamine require further investigation, the results showed that the applied constraints were able to predict a range of metabolic processes that were subsequently observed in the experimental data from the HuMiX cocultures. In addition, a predicted core set of metabolites and formate may play a role in fusobacterial-host interactions, not only in in vitro cocultures, but also in CRC patients. In our next experiments, we continued to use the well-described CRC-associated bacterium *Fn* as a model to study the mechanisms behind microbe-host crosstalk in the context of CRC.

### Formate increases cancer stemness, driving CRC cell invasion

**Fig. 4:**
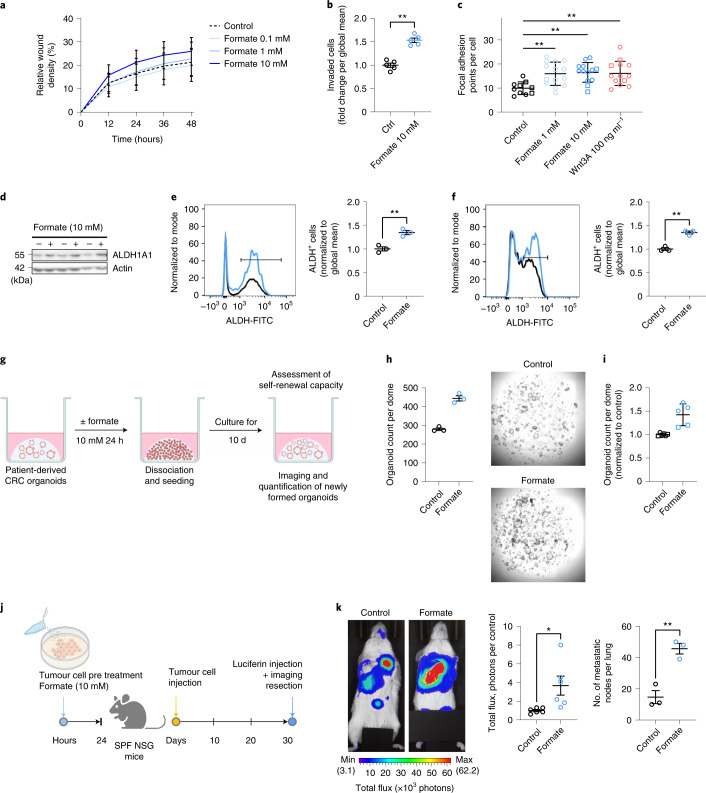
Formate drives CRC cell invasion by increasing cancer stemness. **a**, Scratch wound healing capacity of HCT116 CRC cells over 48 h, *n* = 3 biologically independent experiments. Gut formate physiological dose is considered to be near 10mM. **b**, Transwell invasion of HCT116 CRC cells at 48 h. Data show the means of replicates from *n* = 5 independent experiments, *P* = 0.0079, two-tailed Mann–Whitney test. **c**, Focal adhesion formation in HCT116 CRC cells after formate or rhWnt3A exposure for 24 h, Data show means of technical replicates with at least 100 cells per condition from two independent experiments (indicated by different shapes). *P* = 0.0150, *P* = 0.0037 and *P* = 0.0067 for formate 1 mM, formate 10 mM and rhWnt3A versus control, respectively, ordinary two-way ANOVA with Tukey’s multiple comparisons test. **d**, ALDH1A1 protein levels in formate (+) treated versus untreated (−) HT-29 cells. **e**,**f**, ALDH activity in human (**e**) or APCmin mouse colonic organoids (**f**) after formate stimulation (5 mM, 24 h; FACS). Left, representative histogram of the ALDH^+^ cell population (crossbar). Right, quantification of ALDH activity, *n* = 3 independent experiments. *P* = 0.0053 in **e** and *P* = 0.0129 in **f**, unpaired two-tailed *t*-test. FITC, fluorescein isothiocyanate. **g**, Schematic representation of experimental setup. Derived control or formate-treated organoids from a CRC patient were dissociated and reseeded at 24 h. **h**, The organoid formation capacity on day 10 after reseeding. Left, the number of organoids counted in one representative experiment out of two, with three technical replicates, Right, representative images of one well per condition, **i**, Normalized organoid formation, *n* = 2 biologically independent experiments (different data point shapes) with two or three technical replicates per condition. **j**, Schematic overview of the intravenous metastatic dissemination model. HT-29-Luc tumour cells were treated with formate before intravenous injection into NSG mice (1 × 10^6^ cells per 200 µl per injection). **k**, IVIS imaging after luciferin injection on day 30 after tumour cell injection. Left, representative images from one mouse per group. Middle, the quantified tumour cell signal from lungs. Data show the total luciferase signal (reported as photon flux per mg of tissue), normalized to control, *n* = 6 biologically independent animals per condition, pooled from two independent experiments. Right, the number of lung macroscopic metastatic nodes, *n* = 3 biologically independent animals per condition from one representative experiment out of two. *P* = 0.0257 and *P* = 0.0045 in the middle and right panels, respectively, unpaired two-tailed *t*-test. **b**, **c**, **e**, **f** and **k** are shown as mean ± s.e.m. *P < 0.05, **P < 0.01. Source data

**Extended Data Fig. 5:**
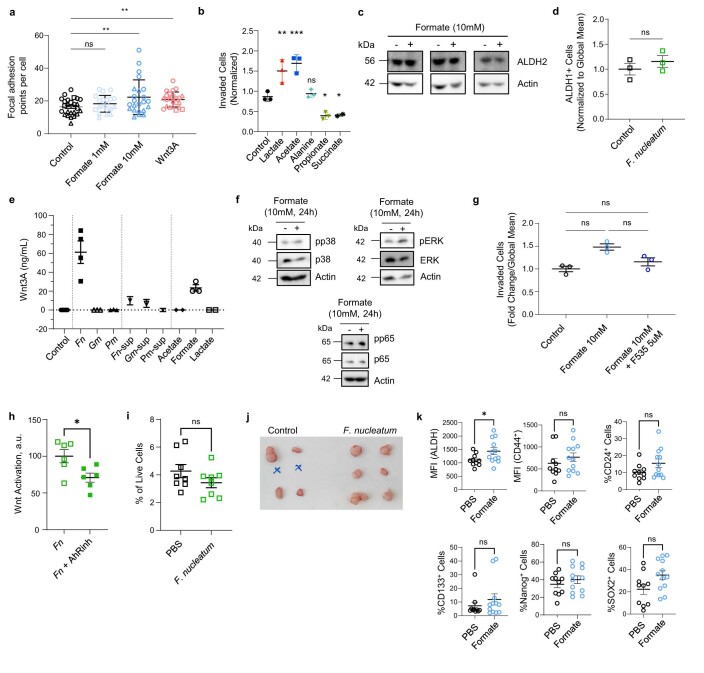
*Fusobacterium nucleatum* and formate induced pathways. **a**, Focal adhesion formation in HT-29 cells after formate or rhWnt3A (100ng/mL) exposure for two days. Data shows technical replicates with at least 100 cells per condition from three independent experiments (different shapes), p=0.0029240 and p=0.0024919 for formate 10mM and Wnt3A vs. control respectively, two-tailed nested ANOVA, with Tukey multiple comparison of means. **b**, Transwell invasion of HCT116 CRC cells after treatment with lactate (positive control), acetate, propionate (10mM), alanine and succinate (5mM), n=three biologically independent experiments, each with three technical replicates, ns=not significant, p=0.0030, p=0.0004, p=0.0217, p=0.0479 for lactate, acetate, propionate and succinate vs. control respectively, ordinary one-way ANOVA Dunnett testing. **c**, Protein levels of ALDH2 in formate-treated (+) and untreated (-) cells. **d**, ALDH activity in human colonic organoids after treatment with *Fn*. Data shows mean±SEM of n=three biologically independent experiments (ns=not significant). **e**, Wnt activity in 7TGO-RKO Wnt-reporter cells stimulated with bacteria *Fn*, *Gemella morbillorum* (*Gm*) and *Parvimonas micra* (*Pm*)) or with 10% [v/v] cell-free bacterial culture supernatants (-sup) or with bacterial secretion products (10mM) for 1h. Bars represent mean±SD of n=one (bacterial supernatants), two (lactate, acetate), three (Gm, Pm, formate, Wnt), four (Fn) or six (control) biological replicates. **f**, Protein levels of pERK/ERK, pp65/p65 and pp38/p38 in formate treated (+) and untreated (-) cells. **g**, Transwell invasion of HCT116 CRC cells after 48 hours of stimulation with formate alone or in presence of a Wnt-signaling inhibitor (F535). Data shows mean±SEM of n=three biologically independent experiments. Control and formate condition alone are also shown in Fig. 4b. ns=not significant, repeated measures one-way ANOVA. **h**, Wnt activity in *Fn*-supernatant treated 7TGP-RKO Wnt-reporter cells alone or with an AhR inhibitor. Data shows mean±SEM of two biologically independent experiments, p=0.0228. **i**, Stemness marker CD44 in mouse tumours after intratumoral injection of *Fn*. Data shows mean±SEM percentage of CD44^+^ live cells (ns=not significant), n=8 independently treated tumors. **j**, Photograph of explanted subcutaneous xenografts from secondary recipient mice from Fig. 5f. **k**, Expression of stemness markers in formate-injected and control tumors, n=10 and n=12 independently treated tumors for the control and formate conditions respectively. Data shows mean±SEM for mean fluorescence intensity (MFI) of ALDH and CD44 and percentage of CD24, CD133, Nanog and Sox2, expressing cells. (ns=not significant, p=0.0460 for ALDH); unpaired, two-sided t-test in (**d**), (**i**), (**j**), and (**l**). *p<0.05, **p<0.01, ***p<0.001. Source data

In our HuMiX experiments, microbiome–host crosstalk was found to induce different signalling pathways associated with cell invasion. Formate was identified as a common and main *Fn* fermentation product (Fig. 2e,f). Since formate is also common to other bacteria^32^ and since endogenous formate was previously shown to affect the invasion of glioblastoma cells in vitro^33^, we proposed that exogenous microbiome-secreted formate could also affect colon cancer cell invasion and ultimately metastatic dissemination. Scratch invasion and transwell assays showed that formate increased the invasion of CRC cells in both experimental setups (Fig. 4a,b). This was accompanied by the formation of a higher number of focal adhesion points per cell (Fig. 4c and Extended Data Fig. 5a). Of the other *Fn*-derived metabolites, only acetate showed an effect on cancer cell invasion, while propionate, succinate and alanine did not (Extended Data Fig. 5b, ref. ^34^). Lactate was used as a positive control for invasion rather than an *Fn-*derived metabolite. Indeed, lactate levels tended to decrease in the HT-29 HuMiX chamber after coculture (Extended Data Fig. 3a).

Tumour metastasis has long been associated with CSCs. Indeed, several studies^35,36^, including one of our own^37^ and a recent one using time-lapse intravital microscopy following cells during dissemination^38^, showed that only cells with CSC activity are able to outgrow into macroscopic metastases. In addition, we observed an increase in genes related to cancer stemness upon coculture with various *Fn* strains (Extended Data Fig. 3f). Therefore, to understand whether the formate-induced invasion of cancer cells could be explained by the acquisition of CSC traits, we first looked for conventional CSC markers such as aldehyde dehydrogenase 1 (ALDH1)^39^. Accordingly, ALDH1A1 protein levels were higher in formate-treated HT-29 cells (Fig. 4d). ALDH2 levels were also slightly induced (Extended Data Fig. 5c). Next, we took advantage of the more physiological colonic organoid model. Treatment with formate resulted in increased ALDH activity in both human organoids (Fig. 4e) and APCmin mouse organoids (Fig. 4f). A similar trend was observed in HT-29 cells upon *Fn* coculture (Extended Data Fig. 5d), altogether suggesting that microbiome-derived formate induces CSC traits in cancer cells. We further used the organoid model to confirm that microbiome-derived formate consequently potentiates the activity and function of CSC. We treated patient-derived organoids with formate for 24 hours. Treated organoids were then single-cell dissociated and reseeded to assess the long-term effects of formate on organoid formation capacity, which is known to be dependent on CSCs (Fig. 4g). After 10 days, we observed enhanced organoid formation upon reseeding formate-treated organoids compared to control organoids (Fig. 4h,i). These results clearly demonstrate that formate induces a higher CSC activity. Finally, we chose the tail vein dissemination assay to validate the formate-induced CSC activity in vivo. This assay focuses on the metastatic outgrowth of cells, which is, as mentioned above, highly dependent on cells possessing CSC traits^35,36,38^. Formate pretreatment of HT-29 cells led to elevated formation of lung metastasis (Fig. 4j,k).

### Formate activates the AhR signalling pathway

**Fig. 5:**
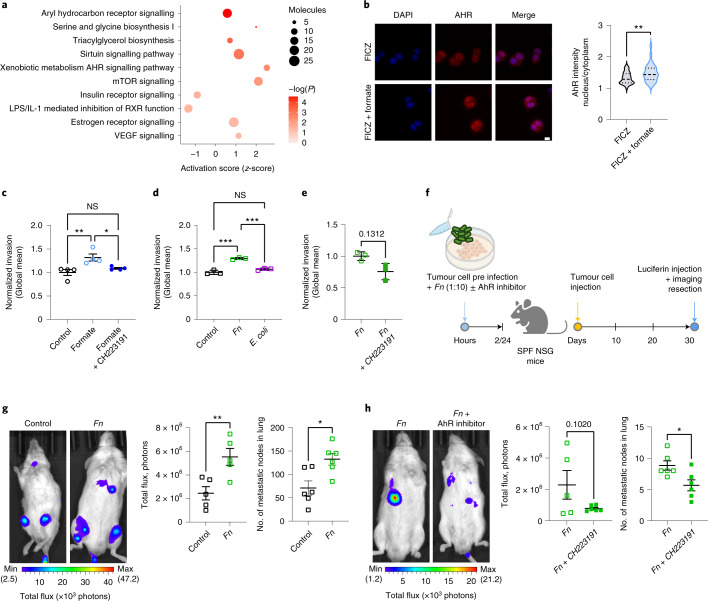
Microbiome-derived formate drives metastatic dissemination through the activation of the AhR signalling pathway. **a**, IPA analysis of differentially expressed genes in formate (10 mM) versus PBS treated HT-29 cells. Plot shows *z*-scores, *P* values and the number of molecules per selected significant pathway (−log(_*P*_) > 1.3), Fisher’s Exact Test. **b**, AhR nuclear translocation in HT-29 cells treated with FICZ (known AhR ligand, 100 nM) alone or combination with formate (10 mM) for 6 h. Data shows technical replicates from two independent experiments, *P* = 0.00113, two-tailed nested ANOVA. Dashed lines represent the medians and dotted lines represent the upper and lower quartiles. **c**, Transwell invasion of HCT116 CRC cells after formate stimulation (10 mM, 48 h) alone, or in presence of an AhR signalling inhibitor (CH223191, 0.5 µM); *P* = 0.0417 for formate versus control; ordinary one-way ANOVA. **d**, Transwell invasion of HCT116 CRC cells after *Fn* and *Escherichia* coli coculture (MOI 10, 2 h). ***P < 0.001; ordinary one-way ANOVA. **e**, Transwell invasion of HCT116 cells upon *Fn* preinfection alone or in presence of an AhR signalling inhibitor (CH223191, 0.5 µM) for 24 h: paired two-sided *t*-test. **c**–**e**, Data show pooled means of replicates from three (**d** and **e**) or four (**c**) independent experiments. **f**, Schematic representation of experimental setup of **g** and **h**: NSG mice were intravenously injected with 1 × 10^6^ cells per 200 µl (**g**) or 0.5 × 10^6^ cells per 200 µl (**h**) HT-29-Luc cells, preinfected with *Fn* (MOI 10) for 2 h and pretreated with (**h**) or without (**g**) AhR inhibitor for 24 h (CH223191, 0.5 µM). After 30 d, signals of HT-29-Luc cells were determined using the IVIS. **g**,**h**, Left, representative image of one mouse per group. Middle, data show the total luciferase signal (reported as photon flux per mg of tissue) normalized to control. Right, the number of lung macroscopic metastatic nodes, *n* = 6 biologically independent animals per condition in **g** and **h**, excluding (**g**) (middle), where *n* = 5 biologically independent animals per condition (one mouse failed luciferin intraperitoneal injection). Regions of interest in **g**: control, 1–11.001 × 10^6^ and *Fn*, 2–3.379 × 10^6^. *P* = 0.0094, *P* = 0.0110 and *P* = 0.0210 in **g** (middle and right) and **h** (right), respectively, unpaired two-tailed *t*-test. Total flux was measured in the chest area. Data in **c**–**e**, **g** and **h** are represented as mean ± s.e.m. *P < 0.05, **P < 0.01, ***P < 0.001. Source data

To better understand the underlying molecular mechanisms of formate-induced CSC activity and metastatic dissemination, we performed RNA-seq on formate-treated HT-29 cells. The IPA analysis revealed that AhR signalling was the most significantly activated pathway (Fig. 5a, *z* = 0.6, *P* < 0.001), similarly to the previous observations in patients bearing *Fn* and in HuMiX (Extended Data Fig. 1e,g, Fig. 2b and Extended Data Fig. 3f). Most of all, AhR not only plays an important role in xenobiotic metabolism, but it also plays a role in the regulation of CSC-like properties via ALDH^40^ or downstream β-catenin/Wnt signalling activation^41^. We therefore proposed the AhR signalling pathway to be responsible for the formate-induced CSC activity described in Fig. 4. Accordingly, formate induced AhR nuclear translocation in HT-29 cells (Fig. 5b). We were able to rescue the effects of formate on CRC cell invasion with an AhR inhibitor (Fig. 5c), suggesting that *Fn*-derived formate induces cell invasion by activating AhR-induced cancer stemness. We propose that this effect is specific to some CRC-associated bacteria such as *Fn*, as Gram-negative *E. coli* did not induce increased tumour cell invasion (Fig. 5d). Concordantly, the pro-invasive effects of *Fn* tended to be rescued by adding an AhR inhibitor (CH223191) in vitro (Fig. 5e). We additionally observed induction of MAPK and Wnt signalling on formate treatment (pathways observed in Fig. 2b in HuMiX and Extended Data Fig. 5e,f), and the formate-induced invasion tended to be inhibited when Wnt signalling was blocked by the small-molecule inhibitor F535 (Extended Data Fig. 5g). It remains unclear how the AhR and Wnt signalling pathways are interlinked in the formate-induced invasion of CRC cells. In breast cancer models, the AhR pathway was described to control CSC proliferation, development, self-renewal and chemoresistance through activation of the β-catenin pathway^42^. Along those lines, *Fn*-induced Wnt signalling was inhibited by AhR inhibition, suggesting that AhR activation can further lead to Wnt activation in our model (Extended Data Fig. 5h).

Finally, we observed a higher metastatic dissemination in the lungs after tail vein injection of cells pretreated with *Fn* compared to controls (Fig. 5f,g). Furthermore, the addition of an AhR inhibitor was able to revert the *Fn*-induced metastatic dissemination (Fig. 5f,h). These results demonstrate that *Fn* induces cancer stemness and thereby metastatic dissemination by activation of the AhR signalling pathway.

Altogether, our data indicate that AhR inhibitors might be of potential therapeutic interest in *Fn*-bearing CRC, who present bleaker outcomes than *Fn*-negative CRC patients^21^.

### *Fn* and formate increase cancer stemness in mice

**Fig. 6:**
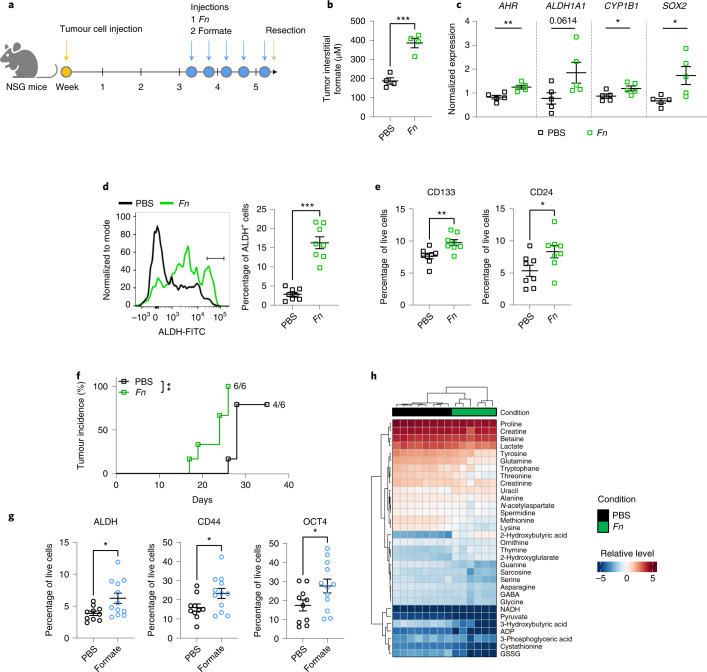
*Fn* and formate increase cancer stemness in vivo. **a**, Schematic overview of the tumour infection mouse model. SPF NSG mice were subcutaneously injected with HT-29 cells subcutaneously (1 × 10^6^ cells per flank). After tumour formation, tumours were injected with *Fn* (MOI 10) or PBS (ctrl) or formate (10 mM, 60 μl) for five consecutive injections over 11 d. **b**, Formate levels in TIF of *Fn*-injected tumours. TIF volume was only sufficient for processing from *n* = 4 independently treated tumours per group. **c**, Gene expression levels of *AHR* (far left), *ALDH1A1* (left), *CYP1B1* (right) and *SOX2* (far right) as assessed by rt–qPCR for *n* = 5 independently treated tumours per group. **d**, ALDH activity assay of *Fn*-/PBS-infected tumours. Left, representative histogram of ALDH^+^ cell populations (crossbar) in mouse tumours assessed by FACS. Right, quantification of ALDH activity in tumours on *Fn* infection. **e**, CD113 (left) and CD24 (right) expression in mouse tumours after intratumoral injection, as assessed by FACS, *n* = 8 independently treated tumours per group in **d** and **e**. **f**, Serial transplantation of *Fn*-treated xenografts. Tumours were explanted at endpoint, dissociated in culture and reinjected subcutaneously in secondary recipient mice at 5,000 cells per flank, *n* = 6 biologically independent animals per group. Kaplan–Meier analysis of tumour incidence was performed using a Mantel–Cox test, *P* = 0.0024. **g**, Expression of ALDH (left), CD44 (middle) and OCT4 (right) in formate-treated and control xenografts as assessed by FACS, *n* = 10 and *n* = 12 independently treated tumours for the control and treated conditions, respectively. **h**, Untargeted metabolite analysis of TIF samples from *Fn*-/PBS-infected xenografts. Heatmap shows normalized intensities, *n* = 8 and *n* = 6 independently treated tumours for the control and treated conditions respectively. Data in **b**–**d** (right), **e** and **g** are shown as mean ± s.e.m, *P* = 0.0006 in **b**, *P* = 0.0051 in **c** (far left), *P* = 0.0482 in **c** (right), *P* = 0.0262 in **c** (far right), ***P < 0.001 in **d** (right), *P* = 0.0052 in **e** (left), *P* = 0.0380 in **e** (right), *P* = 0.0209 in **g** (left), *P* = 0.0409 in **g** (middle) and *P* = 0.0450 in **g** (right), unpaired two-sided *t*-test. *P < 0.05, **P < 0.01, ***P < 0.001. Source data

To evaluate the effects of *Fn*-host cometabolism in vivo, we injected subcutaneous mouse tumours and xenografts with *Fn* (Fig. 6a(1)) or with formate (Fig. 6a(2)). Intratumoral *Fn* injection led to an increase of formate levels within tumour interstital fluids (TIFs) (Fig. 6b), accompanied by an increased *AHR*, *CYP1B1*, *SOX2* and *ALDH1A1* (trendwise) gene expression (Fig. 6c). *Fn* intratumoral administration also led to higher numbers of ALDH^+^ cells in the tumours (Fig. 6d). These results, together with *Fn*-induced expression of CSC markers, such as CD133 and CD24 (Fig. 6e) (with the exception of CD44, Extended Data Fig. 5i), indicate that *Fn* plays a role in CSC regulation, adding to *Fn*-induced metastatic formation observed in colorectal^43,44^ and breast cancer^45^. Of note, we observed that *Fn*-injected xenografts sustained tumorigenic potential in serial transplantation assays, showing increased frequency and shorter latency compared to PBS-injected xenografts (Fig. 6f and Extended Data Fig. 5j). Altogether, these results further support the role of *Fn* in regulating the potential for tumoral self-renewal. To further provide proof that the microbiome-derived formate induces cancer stemness in vivo, we additionally assessed CSC markers after xenograft exposure to formate. Similar to the *Fn* injections, we observed an effect on cancer stemness in tumours injected with formate (Fig. 6g and Extended Data Fig. 5l). Finally, the metabolic profile of the *Fn*-infected TIFs revealed decreased glutamine, lactate and pyruvate levels (Fig. 6h), as previously observed in HuMiX (Fig. 2). Our data therefore indicate that microbiome-derived formate induces CSC activity in tumours.

### *Fn* and formate promote CRC formation and expand Th17 cells

**Fig. 7:**
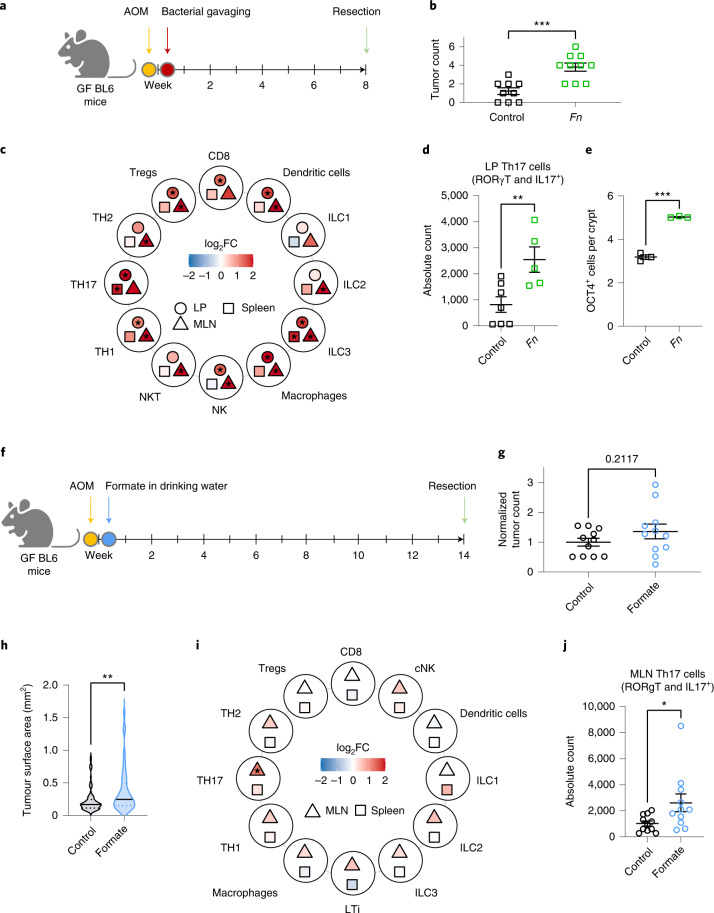
*Fn* administration and formate treatment lead to an increase in tumour incidence or tumour size and an increase in Th17 cells. **a**, Schematic overview of the experimental setup. Germ-free mice received a single dose of AOM intraperitoneal (10 mg kg^−1^). After 3 days, mice were gavaged with bacteria (10^8^ CFU per mouse) and euthanized at 8 weeks postinjection. **b**, Colonic tumour incidence, *n* = 9 and *n* = 10 biologically independent animals in the control and treated groups respectively, pooled from two independent experiments. **c**, Immune cell phenotyping of *Fn* versus PBS gavaged mice. Heatmap shows estimate (log_2_ fold change (FC)) of normalized immune cell counts in mouse lamina propria (LP), MLNs and spleens. Filled star indicates *P* < 0.05, two-tailed least squared means method, **d**, Th17 cell counts (CD4^+^IL-17^+^RORγT^+^ T cells) in LP from **c**. One representative experiment is shown with *n* = 7 and *n* = 5 biologically independent animals in the control and treated groups respectively, in **c** and **d**. **e**, OCT4^+^ cells per colonic crypt, *n* = 3 biologically independent mice, with at least eight crypts per mouse, **f**, Schematic overview of the experimental setup. Germ-free (GF) mice received a single dose of AOM intraperitoneal (10 mg kg^−1^) and formate was administered via the drinking water (250 mM) for the duration of the experiment (14 weeks). **g**,**h**, Tumour incidence (**g**) and surface areas (**h**) in colons of formate-treated mice and controls. **i**, Immune cell phenotyping of formate-treated mice and controls. Heatmap shows estimate (log_2_ fold change) of normalized immune cell counts in the MLNs or spleens. Filled star indicates *P* < 0.05, two-tailed least squared means method. **j**, Th17 counts (CD4^+^IL^-^17^+^RORγT^+^) in the MLNs from **i**. *n* = 11 biologically independent animals per group from two independent experiments pooled for representation in **g**,**i** and **j**. **h**, One representative experiment with all tumour surface areas from four independent biological animals per condition. The solid line represents the median and the dotted line represents the upper and lower quartiles. Data in **b**,**d**,**e**,**g** and **j** are shown as mean ± s.e.m. *P* = 0.0004 in **b**, *P* = 0.0095 in **d**, ***P < 0.0001 in **e**, *P* = 0.0363 in **j**, unpaired two-tailed *t*-test, *P* = 0.00259 in **h**, two-tailed nested ANOVA (factor mouse nested within condition). *P < 0.05, **P < 0.01, ***P < 0.001. Source data

**Extended Data Fig. 6:**
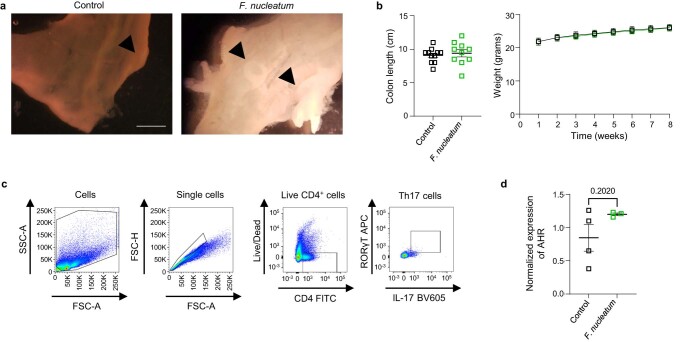
*Fusobacterium nucleatum* and formate induced pathways. **a**, Representative images of open colons of PBS or *Fn* treated mice. Black arrows mark tumors, scalebar=5mm. **b**, Colon length in centimeters (measured upon resection, left) and mouse body weight over time in grams (right) of germ-free mice gavaged with *Fn* and control mice. Data shows mean±SEM, n=11 and n=10 biologically individual animals for the control and *Fn*-treated groups respectively, pooled from two independent experiments. **c**, Representative gating strategy of Th17 cells. Th17 cell gating was performed on live (APC-Cy7^-^) single cells with FITC(CD4)^+^, BV605(IL-17)^+^ and APC(RORγT)^+^ staining. **d**, Gene expression levels of *AHR* as assessed by RT qPCR in *Fn*-gavaged mice. Data shows mean±SEM of n=4 biological replicates in control and n=3 biological replicates in *Fn*, two-sided t-test. Source data

To study the role of *Fn* and its secretion product formate on tumour and immune cells during carcinogenesis, we used a chemically induced germ-free CRC mouse model and administered *Fn* via oral gavage (Fig. 7a). Significantly higher tumour counts were observed after *Fn* gavage (Fig. 7b and Extended Data Fig. 6a) compared to the gavage control, without effects on colon length and mouse weight (Extended Data Fig. 6b). These results were in line with data on similar mouse models^42^. *Fn* led to the expansion and activation of a range of proinflammatory immune cell types (for example, IL4-producing Th2 cells or ILC3s) (Fig. 7c and Extended Data Fig. 6c for the gating strategy). In parallel, we observed an expansion of the regulatory T cell (Treg) compartment upon fusobacterial gavage. An expansion of Treg populations in the spleen of *Fn*-injected mice has been previously described^46^ and shown to correlate with *Fn* in patients, but this phenotype has not been previously observed in the lamina propria^47,48^. Lamina propria NK cell levels were increased after *Fn* gavage (Fig. 7c). A similar effect of *Fn* on NK cells has also been observed following subcutaneous chamber infections^49^. Notably, an expansion of CD4^+^IL-17^+^RORγT^+^ T cells was observed in the lamina propria of *Fn-*treated tumour bearing mice (Fig. 7d and Extended Data Fig. 6c). We also observed a higher number of OCT4-positive cells in the colons of *Fn*-treated mice compared to the control (Fig. 7e), indicating increased cancer stemness, potentially driven by AhR (Extended Data Fig. 6d).

We subsequently used a chemically induced germ-free mouse model (to avoid bias introduced by host microbiome formate production) and administered formate via the drinking water (Fig. 7f). Although tumour incidence was not significantly increased on formate treatment (Fig. 7g), average tumour size was higher compared to that of the control group (Fig. 7h). Similar to the *Fn* administration, formate treatment led to an expansion of CD4^+^IL-17^+^RORγT^+^ T cells in the mesenteric lymph nodes (MLNs) of formate-treated, tumour bearing mice (Fig. 7i,j), suggesting that the effect of *Fn* on Th17 cells is mediated via formate.

## Discussion

**Fig. 8:**
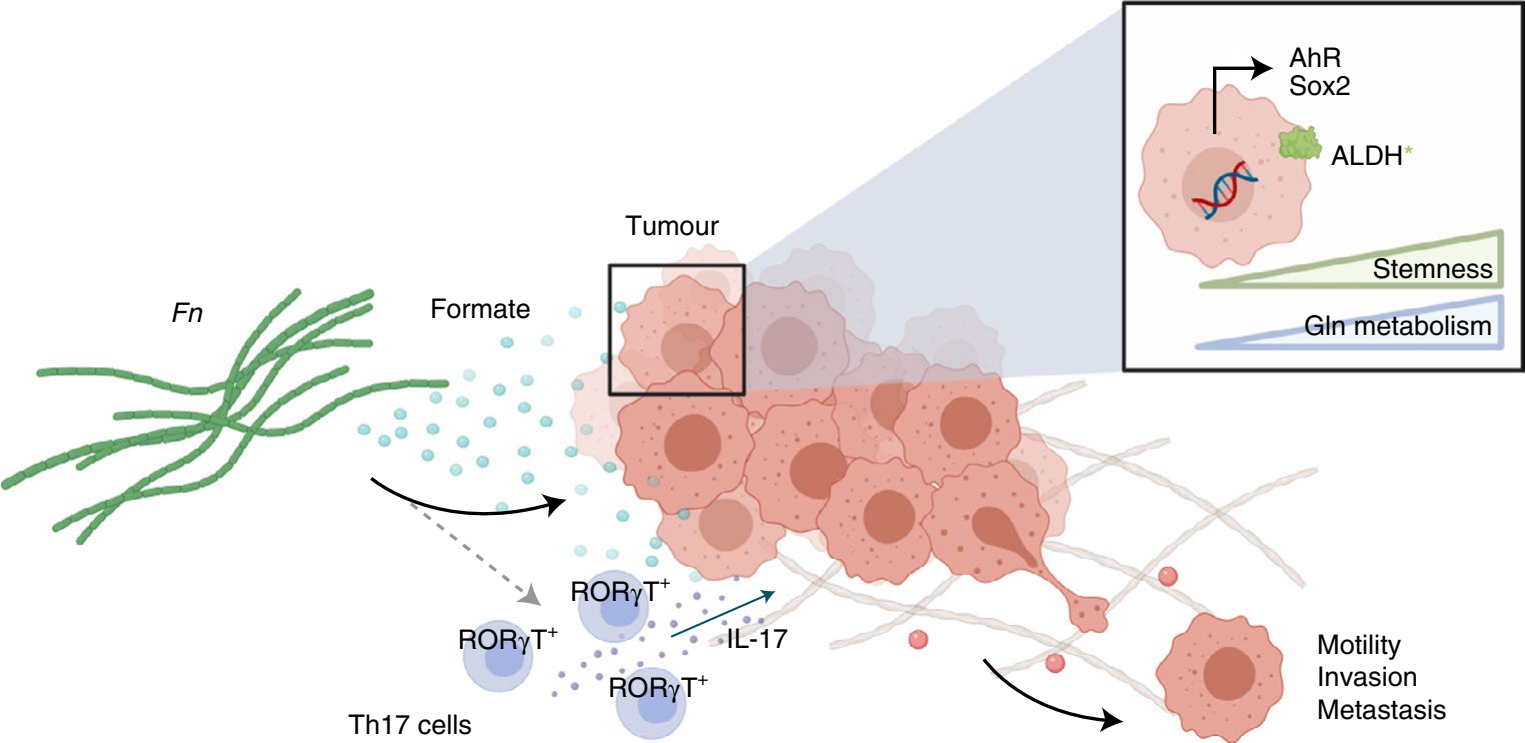
Microbiome-derived formate drives CRC invasion via secreted formate. The *Fn* metabolism product formate promotes AhR signalling in vitro and in vivo, which results in increased Th17 cell infiltration and ALDH activity, cancer stemness and metastatic dissemination of tumour cells.

In this study, we investigated the role of microbiome–host metabolic crosstalk in CRC by using *Fn*, a CRC-associated bacterium, as a model system. Using a metagenomics approach, we showed that tumours with high levels of *Fusobacterium* display an aberrant regulation of genes involved in the metabolism of tumour cells. Using in silico, in vitro and in vivo approaches, we identified formate as a bacterial oncometabolite involved in CRC progression (Fig. 8, graphical abstract).

Our HuMiX-based cometabolism experiments revealed a metabolic shift in tumour cells towards an increased glutamine metabolism. Metabolic reprogramming through increased glutamine metabolism can contribute to cancer therapy resistance^48,49^, and more invasive and metastatic cancer cells tend to be highly glutamine-dependent^50^. Given these findings, the effect of *Fn* on chemoresistance^51^ might additionally be driven by altered glutamine metabolism. We observed increases in pyruvate, succinate and alanine metabolism in *Fn* upon coculture with tumour cells, as well as a reduction in a range of amino acids, suggesting fusobacterial use. An increased capacity of the microbiota to use amino acids via the putrefaction pathway resulting in polyamines, has been shown to promote colon carcinogenesis^52^. Yachida and colleagues observed increased levels of isoleucine, phenylalanine, tyrosine, valine in early-stage CRC^31^, presenting a potential niche to attract *Fusobacteria* and other microbes during these stages. Along this line, besides a depletion of fibre degradation genes, CRC is marked by the enrichment of genes for the use of host carbohydrates and amino acids, together with higher levels of *Fusobacteria*, *Peptostreptococcus*, *Porphyromonas* and others^53^. Of note, lysine fermentation, along with reduced butyrate production, was recently shown to be *Fn*-linked in CRC patients^54^.

Most importantly, we observed elevated levels of formate in our HuMiX coculture experiments. Higher formate levels have previously been associated to chemotherapy non-responders in lung cancer, along with some bacterial species such as *Leuconostoc lactis* and *Eubacterium siraeum*^55^. Thus, the effect of microbial changes on CRC chemoresistance^56,57^, such as increased *Fn*^51^, could be driven by formate. Furthermore, we performed HuMiX experiments using different strains of *Fn* and observed a similar increase in formate levels upon coculture, independent of the strain used. Of note, *Fn* 7_1 is a clinical isolate and showed the highest upregulation in formate upon coculture, underlining the clinical relevance of our findings. This is further supported by a recent study showing high formate production by a clinical isolate of *Bacteroides faecalis*^58^.

The integral results of our in vitro and in silico approaches suggest that a glucose-rich environment allowed for an increased short-chain fatty acid (SCFA) production by *Fn*, when in coculture with tumour cells, with formate as a major metabolic product. Since highly vascularized tumours gain more access to glucose from the bloodstream, the endothelial response in relation to *Fn* could favour formate metabolism. Along this line, studies have described the release of vascular endothelial growth factor in response to *Fn*^59^. In addition, fusobacterial FadA binds VE-cadherin on endothelial cells, and causes VE-cadherin relocation from cell–cell junctions^60^. The resulting increased endothelial permeability facilitates bacterial translocation and can ultimately lead to a hyperpermeable state, a hallmark of pathological angiogenesis found in tumours^61^. Higher tumour vascularization promotes oxidative metabolism, which can lead to a formate overflow in cancer cells^33^, in addition to enhanced secretion of bacterial formate. Indeed, our in vitro and in vivo data hint at increased one-carbon metabolism, marked by serine consumption (Fig. 2d and Extended Data Fig. 3d). This is in line with a recent study showing elevated expression of MTHFD1L, an enzyme involved in formate production and one-carbon metabolism, in CRC patients^62^. Metagenomics data from CRC patients supported the hypothesis of an elevated formate metabolism via pfl along with a *Fusobacterium*-specific protumorigenic metabolic footprint. Nevertheless, the association of pfl activity and formate levels in *Fusobacterium*^high^ patients requires further analysis by using formate-deficient mutants in vitro and in vivo. Of note, *Fusobacterium* is significantly tumour-enriched, which may explain its role in specifically increasing formate at the tumour site.

Previous studies on metagenomics data have revealed bacterial formate oxidation as an overrepresented metabolic pathway in a chemically induced colitis mouse model^32^. Formate is known to be mainly produced by the gut microbiome, as formate cannot be detected in germ-free animals^32^. It is produced and secreted by several different bacteria^58,63^. In specific pathogen free (SPF) mice, dysbiosis was accompanied by an increase in luminal formate levels in a millimolar range^32^. These metabolic shifts can ultimately bridge the colonization of, and provide fitness advantages to, further opportunistic pathogens (for example, pathogenic *E. coli* strains) possessing formate dehydrogenase and terminal oxidase genes, as shown in murine models of colitis^32^. Furthermore, a recent study associated formate, propionate, and acetate levels to *Faecalibacterium* and *Bifidobacterium* in the faecal microbiomes of inflammatory bowel syndrome patients^64^. Taken together, these studies underline the variety of formate-producing microbes, further extending its importance beyond *Fn*. Nevertheless, the protumorigenic effect of formate might be context dependent and regulated by other metabolic factors in the tumour niche. Further studies are required to explain these complex interactions surrounding formate and its function in the tumour microenvironment.

SCFA have been shown to control important signalling pathways responsible for AhR signalling and stem cell proliferation and maintenance: either as inducers or inhibitors^65–67^. Using various in vitro and in vivo models, we now show that formate is able to induce invasion of cancer cells potentially by inducing AhR signalling and its associated CSC regulation. These results agree with our previous study in which formate increased the tumour-initiating capacity of cancer cells^68^. Taken together, ALDH activity, as well as signalling activation of the genomic and non-genomic AhR pathways with functional consequence for other pathways (β-catenin/Wnt, NF-κB, MAPK and FAK)^40^, indicate that microbiome-secreted formate might play an important role in the malignant transformation of intestinal cells and the control of their stemness properties.

Furthermore, immune cell regulation might be an additional factor in formate-driven tumorigenesis. In line with other previous studies^42^, our in vivo data showed *Fn*-accelerated tumour incidence. In general, we observed a proinflammatory effect of *Fn* through the expansion of CD4^+^IL-17^+^RORγT^+^ T cells in MLNs. It has been shown that activation of AhR during murine Th17 cell development increases Th17 T cell populations and their production of cytokines^69^. Consequently, formate, secreted by *Fn* or other tissue invading microbes, may affect the Th17 T cell compartment in CRC. Accordingly, we observed that supplementation of formate in mice led to an increase in CD4^+^IL-17^+^RORγT^+^ T cells. Hence, AhR inhibitors, which have already been described as a potential new therapeutic target in cancer^70^ may be used for patients with *Fusobacterium*-positive tumours or with elevated levels of formate and formate-producing bacteria.

Altogether, our study offers an integrative approach on studying the role of the microbiota in CRC with a focus on host–microbe cometabolism. A protumorigenic phenotype that comes with enhanced fusobacterial metabolite secretion has not been observed before in the CRC context. The results indicate implications for the role of microbiome-derived metabolites, especially formate, as a key player responsible for *Fusobacterium*’s protumorigenic properties via enhanced AhR signalling and CSC regulation. Of note, our study demonstrates the role of formate in cancer stemness, and in conjunction with other previously discussed studies, it further raises the question of community-wide formate production, not only by *Fusobacterium*, but also by other bacterial species. Altogether, we identified microbiome-derived formate as an oncometabolite in CRC.

## Methods

### Human sample acquisition and DNA extraction

Patient samples were donated willingly under informed consent and were handled in accordance with institutional guidelines. Ethical approval was obtained from the Comité National d’Ethique de Recherche, Luxembourg (reference 201009/09) followed by institutional approval by the Ethics Review Panel of the University of Luxembourg (ERP-16-032). Patients underwent surgery at the Centre Hospitalier Emile Mayrisch (Esch-sur-Alzette, Luxembourg), the Centre Hospitalier de Luxembourg (Luxembourg) or the Zitah Klinic (Luxembourg). A healthy control cohort (ND collection) was enrolled by the National Centre of Excellence in Research on Parkinson’s disease (NCER-PD) and 16S rRNA gene sequencing data were shared according to a biomaterial cooperation agreement. The average ages of stool sample donors were 70.29 ± 8.74, with 12 female and 40 male participants (in-house CRC collection) and 62.05 ± 7.51, with 30 female and 33 male participants (ND collection). All patients were enrolled between the years 2010 and 2019. No participant compensation was provided. Stool samples were collected in OmniGut tubes (DNA Genotek) at the time of surgery (patients with CRC) or at home (healthy donors). Patients received a mild phosphate enema the day before surgery. Metadata can be found in (Supplementary Table 1).

Primary colon cancer tissue and matched distant non-neoplastic colon tissue (at the farthest longitudinal surgical margin) were collected following the standard preanalytical practices for biospecimens, established by the Integrated Biobank of Luxembourg (IBBL). Immediately after surgery, fresh samples were transported to the laboratory on ice for further downstream processing (generation of primary cell cultures and organoids within one hour after resection). A pathologist from the National Center of Pathology, Laboratoire National de Santé provided the clinical and histopathological data.

RNA and DNA were extracted from stool or tissue with the AllPrep kit for RNA, DNA and protein extraction (Qiagen).

### 16S rRNA gene sequencing analysis

16S rRNA gene sequencing was performed at the IBBL. Sequencing of the V3 and V4 regions of prokaryotic 16S rRNA gene was performed on the Illumina MeSeq platform using 2 × 300 bp paired-end reads. Raw 16S rRNA gene sequences (FASTAQ files) were cleaned and clustered as operational taxonomic units using NgTax or the Dada2 pipeline. Bacterial abundances were plotted as base means.

### Bacterial strains and growth conditions

Bacterial strains were obtained from the German Collection of Microorganisms and Cell Cultures (DSMZ) or through collaborations and are listed in Supplementary Table 5. Adjusted from the DSMZ culture recommendations, bacterial strains were revived from glycerol stocks in 3 ml of anoxic brain heart infusion broth (Merck). Bacterial inoculums were maintained in an anaerobic chamber at 37 °C, 95% N_2_ and 5% CO_2_.

### Cell lines

HCT116, HT-29 and Caco-2 CRC cell lines were obtained from ATCC and maintained in DMEMF12 with 10% [v/v] foetal bovine serum (FBS) and 1% [v/v] penicillin/streptomycin. RKO-7TGP from the University of Vienna (Dolznig laboratory) were maintained as previously described^71^. HT-29 cells were obtained from ATCC. Firefly luciferase-green fluorescent protein (GFP) lentiviral particles (PLV-10172-50, Cellomics Technology) were used to transduce HT-29 cells (HT-29-Luc) at 1 × 10^8^ TU per ml per followed by puromycin selection. Cell lines (purchased from ATTC) were frequently checked to be mycoplasma-free and were authenticated before and after their use in this study (STR analysis, DSMZ).

### Primary organoid generation

Muscle and mucus were removed from tissue samples (human or C57BL/6J-*Apc*^*Min*^/J mouse colon) using surgical scissors. The tissue was cut into small pieces and washed 3–4 times with cold PBS until all visible debris was removed. The tissue pieces were resuspended in 5 ml of Gentle Cell Dissociation Reagent (STEMCELL Technologies) and placed at 4 °C on a benchtop roller for 30–60 min. After incubation, the tissue pieces were washed three times in 10 ml of cold PBS (vigorous shaking) and discarded. The washes were filtered through a 70-μm pore-sized filter and crypts were resuspended at 300 crypts per 20 µl of Matrigel matrix per dome (Corning) and seeded into 48-well plates. Matrigel was left to solidify for 10 min at 37 °C, then 250 µl of Human or Mouse Organoid Growth Medium (hOGM/mOGM, STEMCELL Technologies) with 10 µM Y-27632 (STEMCELL Technologies, catalogue no. 72304) was added to each well. Organoids were passaged after 7–14 d with total medium changes (without Y-27632) every 3–4 d. Organoids were passaged using Gentle Cell Dissociation Reagent and reseeded as described above at a ratio of 1:1.5–1:2.

### HuMiX cocultures

The HuMiX device is a modular bioreactor perfusion system allowing for the coculture of bacteria and human cells, as described previously. HuMiX devices are assembled and primed 1 d before the beginning of the experiments. In short, the top chamber was perfused with N_2_ gas (0.1 l min^−1^ flow rate), establishing anoxia (roughly 0.4–0.6% O_2_). It was separated from the underlying microbial chamber by a nanoporous membrane (0.05 µm) coated with porcine mucin (Sigma-Aldrich), dissolved in ddH_2_O (0.025 mg ml^−1^). Another nanoporous membrane separated the microbial chamber from the underlying epithelial chamber, which in turn was coated with rat-tail collagen solution (50 µg ml^−1^, Corning). A microporous membrane (1.0 µm) separated the epithelial chamber from the bottom chamber, which supplied fresh oxic (roughly 6% oxygen) growth medium to the human cells. The constant perfusion flow rate was 25 μl min^−1^ (0.5 r.p.m., peristaltic pump). Epithelial cells (HT-29, Caco-2) were inoculated 6 d before bacterial inoculation (*Fn* 25586, *Fn* 23726 and one clinical isolate *Fn* ssp. *animalis* 7_1). Bacterial–human coculture lasted 24 h before the devices were opened and supernatants and cells were collected for downstream analysis (RNA, DNA, proteins, extracellular metabolites). Control devices with bacterial or human cell monocultures were run in parallel.

### RNA-seq

For library preparation, 1 µg of RNA was poly(A) selected, fragmented and reverse transcribed by using the Elute, Prime, Fragment Mix (Illumina). End repair, poly(A)-tailing, adaptor ligation and library enrichment were performed as described in the Low Throughput protocol of the TruSeq RNA Sample Prep Guide. RNA library quantity and quality were assessed with the Agilent 2100 BioAnalyzer (Agilent). RNA libraries were sequenced as 100 bp paired-end runs on an Illumina HiSeq2500 platform.

### Metabolomics

Before untargeted semiquantitative GC–MS analysis (Agilent MassHunter, v.B.08.00), sample derivatization was carried out using an automated sample preparation robot in two steps: methoxamine hydrochloride ketone stabilization and *tert*-butyldimethylsilyl group replacement. After derivatization, we carried out GC–MS analysis in an Agilent 7890A GC coupled to an Agilent 5975C inert XL Mass Selective Detector. For precision quantification, GC–MS measurements of selected derivatives were performed using a selected ion monitoring mode. The automated in-house designed Metabolite Detector software, in combination with an in-house library, were used for peak assignment of all chromatographs and metabolites^72^.

For highly volatile short chain fatty acid detection (targeted and absolute quantitative), the derivatization was performed in the organic phase using diethyl ether. A dilution series of a volatile free acid mix was prepared for the quantitative measurement. Then 10 μl of 200 mM ethylbutyric acid (internal standard), 1 ml of 99% diethyl ether and 10 μl of 1 mM hydrochloric acid were added to each reaction tube primed with 190 μl of fluid (fresh medium, ultrapure water as blanks, for each time point of the dilution series). The mix was agitated for 10 min at room temperature and centrifuged at maximum speed for 5 min at 15 °C. The upper phase was transferred to a new 2-ml reaction tube. Then 1 ml of diethyl ether was added to the tube with the lower phase and a second agitation/centrifugation/transfer step was performed. 250 µl of each sample were then distributed into GC vials in triplicates and 25 μl of MTBSTFA (with 1% *tert*-butyldimethylsilyl chloride) were added to them. Finally, the samples were analysed using GC–MS. Quantification of metabolite levels in mouse TIF by liquid chromatography–mass spectrometry as previously described (minimal volume 50 μl required, samples with yields under 50 μl were not processed, Xcalibur software, v.4.3.73.11)^73^. All resulting raw data were analysed in R.

### Western blot

Cells were washed with 4 °C PBS and lysed with 4 °C RIPA buffer containing 1% SDS and protease inhibitor. After addition of Lämmli buffer, lysates were vortexed and supernatants were heated to 95 °C for 5 min. Then 20 μg of proteins were resolved by SDS–PAGE on 12% gels, transferred onto a polyvinylidene difluoride–PLUS Transfer Membrane (0.2 µm). Blocking was performed with TBS-N + 10% BSA for 1 h at room temperature, followed by an overnight incubation at 4 °C for probing with specific primary antibodies (MAB1501, Millipore and sc-32293, Santa Cruz at 1:5,000; 12035, 18818S, 4370S, 4694, 9154S, 9212, 4511, 3033T, Cell Signalling and sc-93, sc-109, Santa Cruz at 1:1,000) diluted in TBS-N + 5% BSA. Membranes were washed three times with TBS-N and incubated for 1 h at room temperature with the corresponding horseradish peroxidase (HRP)-labelled secondary antibody (7076s and 7074 at 1:500). The blots were revealed using enhanced chemoluminescence on a Fusion FX imaging platform.

### Real-time quantitative PCR (qPCR)

RNA extractions, reverse transcriptions and real-time qPCRs were carried out according to previously reported protocols^28^. Reference genes used for qPCR were *ACT1* and *GAPDH* (for mouse) and *YWHAZ* and *EEF1A1* (for human). Primer pairs used for qPCR with reverse transcription (RT–qPCR) are listed in either Supplementary Table 5 or described previously^28^. rt–qPCR was analysed using qBase+ v.3.2 (Biogazelle) according to The Minimum Information for Publication of Quantitative Real-Time PCR Experiments guidelines.

### Reporter gene assay

Here, 50,000 7TGP-RKO β-catenin/Wnt signalling reporter cells were seeded in 24-well plates. Bacterial suspensions were added to the cell growth medium at a multiplicity of infection (MOI) of 1:20 for 1 h. Cells were then washed twice with medium, supplemented with 1% [v/v] penicillin/streptomycin and further incubated in antibiotic-containing media for 48 h. For supernatant, formic acid 10 mM + 1% [v/v] 1 N NaOH or control treatments, 10% [v/v] cell media was supplemented accordingly and cells were incubated for 48 h. After trypsinization, cells were stained with LIVE/DEAD Fixable Near-IR Dead Cell Stain (ThermoFisher) and viable GFP^+^ cells were acquired by fluorescence-activated cell sorting (FACS). Standards of rhWnt3A protein were used.

### AhR nuclear translocation assay

HT-29 cells were treated with 6-formylindolo(3,2-b)carbazole (FICZ) alone (100 nM), or FICZ (100 nM) and formate (10 mM) for 6 h. After treatment, cells were fixed in 4% paraformaldehyde at room temperature for 10 min and permeabilized with 0.1% Triton-X. Cells were stained with 1:500 dilutions of a polyclonal anti-AhR antibody (Enzo Life Sciences, BML-SA210), followed by donkey anti-rabbit AF647 secondary antibody (Thermofisher, A-31573). Nuclei were visualized using 4,6-diamidino-2-phenylindole (DAPI). Fluorescence micrographs of cells were obtained with a fluorescent microscope (OLYMPUS IX83) at ×60 magnification. Intensity of AhR staining in nuclei and cytoplasm was assessed using ImageJ, and ratio of AhR intensity between nucleus and cytoplasm was calculated for single cells.

### Scratch wound invasion assay

Cancer cell lines were seeded on rat-tail collagen type I-coated (200 μg ml^−1^) 96-well Imagelock plates (Sartorius) at approximately 90% confluence in cell culture medium containing rat-tail collagen type I (1 mg ml^−1^). A wound was scratched in the cell monolayer across each well with the CellplayerTM 96-well WoundMaker (Sartorius). Confluence of the wound area was monitored with the IncuCyte imaging system (Sartorius) every 3 h for a total of 72 h. At the end of the experiment, the IncuCyte ZOOM software (2018B, Sartorius) was used to calculate a relative wound density for each time point by measuring the spatial cell density in the wound area relative to the spatial cell density outside the wound area.

### Transwell invasion assay

Here, 50,000 HCT116 cells were seeded in serum-free medium in the upper compartment of ThinSert transwells (8 µm, Greiner). Medium containing 10% FBS [v/v] was added to the bottom compartment as a chemoattractant. Cells on transwells were treated with formate (10 mM) ± AhR inhibitor (CH223191, 0.5 µM), or preinfected with *Fn* (MOI 1:10) for 2 h, washed twice with medium, supplemented with 1% [v/v] penicillin/streptomycin and further incubated in antibiotic-containing media for 24 h ± AhR inhibitor, at 37 °C for 48 h. Cells were fixed with 4% PFA and stained with the cytological dye crystal violet (0.125% [v/v] in PBS; Sigma-Aldrich). Residual non-invasive cells were manually removed from the apical membrane side with a cotton swab. The transwells were dried overnight at room temperature and examined under a microscope. ImageJ software was used to and measure contrast-based cell confluence of treated cells relative to control (untreated) cells.

### Focal adhesion assay

Here, 50,000 cells were seeded on 1 cm Ø glass cover slips in 12-well plates and treated with formate (1 or 10 mM) or rhWnt3A (100 ng ml^−1^, PeproTech) for 48 h. Cells were fixed in 3.5%PFA for 30 min, washed with PBS and PBS-Trition 1%, blocked with 5% milk and washed again in PBS-Triton 0.5%. Focal adhesions were stained overnight with mouse-anti-Vinculin (primary antibody; V4505, Sigma-Aldrich), before being further washed with PBS-Triton 0.5% and stained with goat-anti-mouse-DyLight488 (ab96879, Abcam), Phalloidin AlexaFluor594 (A12381, ThermoFisher) and DAPI (ThermoFisher). Pictures were randomly taken under a confocal microscope and images were analysed using ImageJ (version 1.53K).

### Organoid assay

Organoids embedded in Matrigel were treated with 5 mM sodium formate (Carl Roth, 4404.1), for 48 h at 37°C. ALDH activity was assessed according to the manufacturer’s protocol (ALDEFLUOR Kit, Stemcell Technologies, 01700). CD133 (Miltenyi Biotec, 130-090-826), CD44 (BD Biosciences, 555428) and CD24 (BD Biosciences, 560533) labelling was done at a 1:50 dilution. LIVE/DEAD cell viability staining was performed at a 1:1,000 dilution (Invitrogen, L34975). Samples were acquired on a FACS CantoII Cell Analyzer (BD Biosciences). Results were analysed with the FlowJo software (BD Biosciences).

For functional assays, organoids derived from patients with CRC embedded in Matrigel (Corning, catalogue no. 356231), were treated with 10 mM sodium formate (Carl Roth, catalogue no. 4404.1) or PBS as a control in hOGM (STEMCELL Technologies, catalogue no. 06010) for 24 h at 37 °C. Matrigel domes were dissociated and reseeded as described above, at 40 × 10^3^ dissociated single cells per well. Organoids were maintained for 10 d as described above. Plates were imaged with Cytation 5 (BioTek). Organoids were counted using ImageJ.

### Constraint-based modelling

The microbial reconstructions from the AGORA resource^29^ were downloaded from the Virtual Metabolic Human website (www.vmh.life)^74^. The models were manually curated and analysed through functions implemented in the COBRA 3.0 Toolbox^75^ using IBM ILOG Cplex (IBM, Inc.). Pairwise models were built and interrogated using the pairwise modelling module in the Microbiome Modelling Toolbox^76^. The *Fn* model for the interaction analysis was curated on the basis of the Fusobacterium_nucleatum_subsp_nucleatum_ATCC_25586 AGORA model (version November 2019 from vmh.life; addtoFNmodel.m script: addition of 18 reactions, for example for the uptake and hydrolysis of dipeptides, the production of *N*-acetylputrescine or the transport of lactate and 2-hydroxybutyrate). The simulations were performed as previously described^68^. The HT-29 model was set up as a conditioned, cell type specific model on the basis of RNA-seq gene expression data of HT-29 monocultures in HuMiX with a Recon 2.0 background as previously described^6^.

### Animal models

All animal experiments were performed according to all applicable laws and regulations (EU Directive 2010/63/EU and Grand-Ducal Regulation of 11 January 2013 on the protection of animals used for scientific purposes), after receiving approval from the Animal Experimentation Ethics Committee at the University of Luxembourg (AEEC) and the Ministry of Agriculture, Viniculture and Rural Development (LUPA 2019/99 and LUPA 2019/60). Mice were housed in a SPF or a germ-free facility at a relative humidity of 40–70%, at 22 °C and in 12 h dark/light cycles. They received food (SAFE A40) and water ad libitum.

### Dissemination/metastasis model (SPF)

Here, 7–8-week-old in-house bred male and female nod scid gamma (*Mus musculus* NSG) mice were intravenously injected with 0.5–1 × 10^6^ per 200 µl of HT-29-Luc cells, pretreated with or without 10 mM formate (24 h) or preinfected with *Fn* (MOI 1:10) for 2 h, washed twice with medium, supplemented with 1% [v/v] penicillin/streptomycin and further incubated in antibiotic-containing media for 24 h ± AhR inhibitor (CH223191, 0.5 µM). After 30 d, mice were intraperitoneally injected with luciferin (15 mg ml^−1^, 100 µl per 10 g mouse weight). Then 10 min postinjection, signals were detected using the In Vivo Imaging System (IVIS). Macroscopic counting of metastasis nodes of the lungs was performed blindly.

### Xenograft infection models (SPF)

For this, 7–8-week-old in-house-bred male and female nod scid gamma (*M. musculus* NSG) mice were subcutaneously injected with 1 × 10^6^ HT-29 tumour cells. When tumours reached a size of approximately 200 mm^3^, 5 × 10^7^ *Fn* ATCC 25586 or formate (10 mM) was injected intratumorally (three injection sites per tumour) every other day for 10 d (for a total of five injection time points). Xenografts were harvested at endpoint, weighed, subjected to TIF extraction as previously described^73^ and dissociated (Human Tumour Dissociation Kit, Miltenyi) for further FACS-based protein expression analysis. For serial transplantation of Fn-injected xenografts, primary tumours were explanted, dissociated in culture and reinjected in secondary recipient mice, as previously described^28^. Tumour incidence and growth was monitored over time blindly by palpation of the flanks. The maximal total tumour volume of 2,000 or 1,500 mm^3^ per single tumour permitted by the AEEC was not exceeded.

### Treatment models (bacteria and formate in germ-free)

Here, 11–15-week-old germ-free in-house-bred C57BL/6Ntac mice (*M. musculus*) received a single 10 mg kg^−1^ intraperitoneal azoxymethane (AOM) injection (A5486, Sigma-Aldrich). Three days later, male and female mice received 10^8^ colony forming units (CFU) *Fn* suspended in 200 µl of PBS via by oral gavage (PBS alone as control) or formate (250 mM) was provided in the drinking water (male mice only) for the course of the experiment (pure drinking water as control)^77^. At endpoint (8 or 14 weeks after AOM injection, for bacterial gavage or formate treatment respectively), mice were euthanized via intraperitoneal injection of medetomidine (1 mg kg^−1^) and ketamine (150 mg kg^−1^). Organs were harvested and snap-frozen in liquid nitrogen on collection and stored at −80 °C until analysis. A 0.5 cm snip of the colon containing a stool pellet was embedded in TissueTek and snap-frozen in liquid nitrogen. Another 0.5 cm snip was directly snap-frozen in liquid nitrogen. The rest of the colon was cut open longitudinally, macroscopic tumours were counted blindly by two independent researchers, and the colon was subjected to immune cell phenotyping as described below. Photographs of the tumours were taken and tumour size was quantified using ImageJ from photographs acquired on a Wild M10 (Leica), at ×8 magnification.

### Immune cell phenotyping

**Extended Data Fig. 7:**
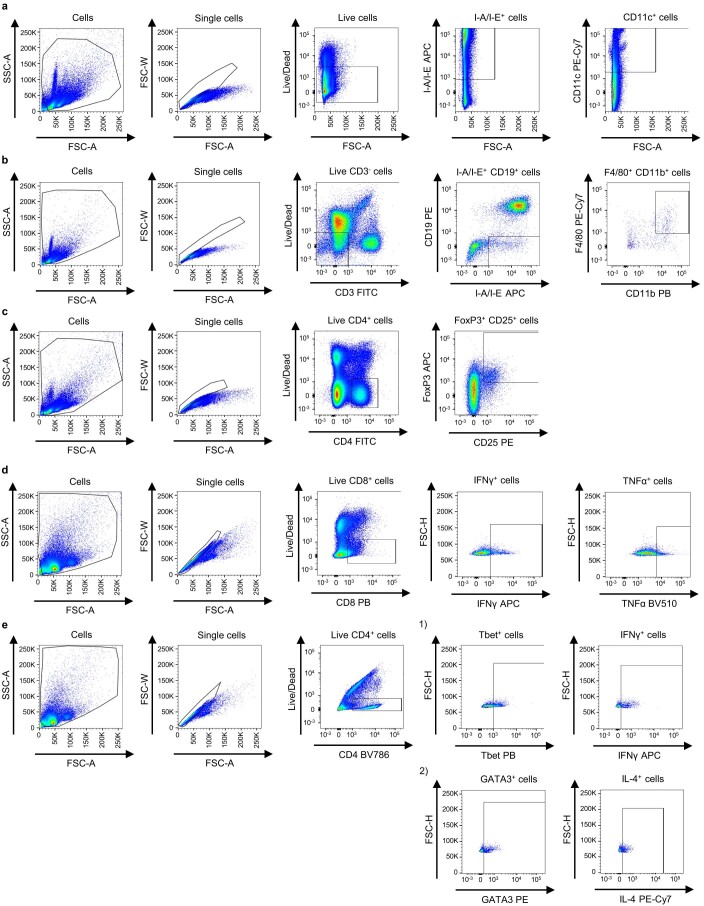
Flow cytometry gating strategies. **a**, Representative gating strategy of dendritic cells. **b**, Representative gating strategy of macrophages. **c**, Representative gating strategy of T regulatory cells. **d**, Representative gating strategy of CD8 cells. **e**, Representative gating strategy of T helper 1 (subpanel 1) and T helper 2 (subpanel 2) cells.

**Extended Data Fig. 8:**
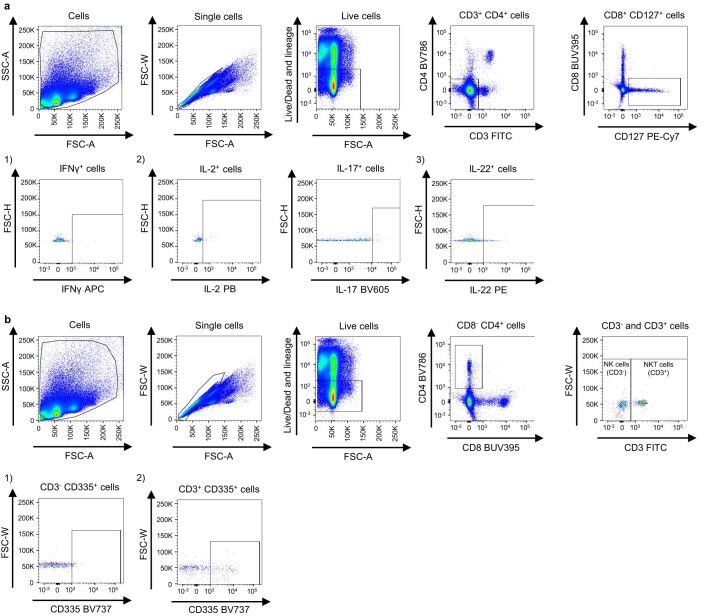
Flow cytometry gating strategies. **a**, Representative gating strategy of innate lymphoid cells (ILC); ILC1 (subpanel 1), ILC2 (subpanel 2), ILC3 (subpanel 3). **b**, Representative gating strategy of natural killer (NK, subpanel 1) and natural killer T (NKT, subpanel 2) cells.

Colons were opened longitudinally, washed with cold PBS, cut into smaller sections and digested first in Roswell Park Memorial Institute medium (RPMI) containing glutamine/GlutamMAX, 3% FBS, 1% P/S, 5 mM EDTA and 0.154 mg ml^−1^ DTT, then in serum-free RPMI with Liberase (0.1 mg ml^−1^) and DNase (0.5 mg ml^−1^), both in a shaking incubator at 800 r.p.m. and 37 °C for 20 and 30 min, respectively. Spleens were digested in serum-free RPMI with Liberase (0.1 mg ml^−1^) and DNase (0.5 mg ml^−1^) for 30 min. Digestions were quenched with RPMI medium containing 10% FBS and cell suspensions were filtered through a 70-µm filter. MLNs were mashed through a 70-µm filter. Single-cell suspensions were restimulated in RPMI containing 10% FBS, 0.1 µg ml^−1^ PMA and 1.5 µg ml^−1^ ionomycin for 4 h at 37 °C. Brefeldin A was added 1 h after the beginning of the restimulation. Cells were stained with Near-IR fixable live/dead dye and with the corresponding antibody panels (Supplementary Table 6) using the Cytofix/Cytoperm kit (554714, BD Bioscience). An additional Fc blocking step preceded antibody staining for macrophages and dendritic cells. Acquisition was performed on a CantoII and a Fortessa FACS machine and data were analysed using BD FACSDiva v.8.0.1 and FlowJo software v.10.6.1 (gating strategies can be found in Extended Data Figs. 7 and 8). Total cell counts were determined by Cedex and multiplied by frequency of subpopulations to get absolute counts.

### Animal tissue sections and staining

Animal tissues were embedded in TissueTek (Sakura) and snap-frozen in liquid nitrogen immediately on collection. Cryosections were fixed with ice-cold methanol for 10 min at −20 °C, blocked with a 3% BSA solution and incubated overnight at 4 °C with primary antibodies (Supplementary Table 5). Secondary antibodies (Supplementary Table 5) were applied for 1 h at room temperature. Sections were mounted with DAPI Fluoromount-G (ThermoFisher), viewed under a confocal microscope and processed with ImageJ.

For OCT4 staining, 7-μm cryosections were fixed in acetone and stained with 1:500 dilutions of anti-OCT4 (primary antibody, Abcam, ab18976) and goat anti-rabbit AF647 (secondary antibody, Invitrogen, A-21245). Sections were mounted using DAPI Fluoromount-G (SouthernBiotech, 0100–20). Images were acquired using a Cytation 5 microscope (BioTek Instruments) and analysed using ImageJ.

### Computational tools, data and statistical analysis

R studio (v.1.2.5042, R studio Team 2020) with R CRAN (v.4.0.3, R Core Team 2020) was used for the analysis of 16S rRNA gene and RNA/WX sequencing, as well as for metabolomics data analysis. Plots were made using ggplot2. MATLAB (R2018B) and the COBRA toolbox (3.0) were used for the constraint-based modelling. PathSeq analysis was conducted as described in^22^. 16S and RNA-seq data were analysed using the dada2. RT–qPCR data were analysed using qBase+ 3.2 (Biogazelle). We used the CMS subtypes published by Guinney et al. for TCGA samples. As EGA samples were not characterized in the Guinney report, we used the R CMScaller package to characterize the subtypes^78^. Data were analysed through the use of IPA (QIAGEN Inc., https://www.qiagenbioinformatics.com/products/ingenuity-pathway-analysis) with input lists of differentially expressed genes, filtered for log_2_ fold change >1.5 and *P* < 0.05. GraphPad Prism or R was used for statistical analysis, which is further described in the respective figure legends.

### Reporting Summary

Further information on research design is available in the Nature Research Reporting Summary linked to this article.

## Supplementary information

Reporting Summary

Supplementary Tables Supplementary Tables 1–6.

## Data Availability

Sequence data from the EGA are available under accession number EGAS00001000288. Sequence data from the patients with CRC and healthy donor samples can be found on ENA (EBI) under the accession number PRJEB46665. The TCGA analysis was based on data (TCGA-COAD, TCGA-READ) generated by the TCGA Research Network: https://www.cancer.gov/tcga. All scripts and models can be found under https://gitlab.lcsb.uni.lu/mdm/ternes_et_al_2022/. Microbial reconstructions are modified from the Virtual Metabolic Human website (www.vmh.life) and available on gitlab. Source data are provided with this paper.

## Source data

Source Data Fig. 1 Numerical source data.
Source Data Fig. 2 Numerical source data.
Source Data Fig. 3 Numerical source data.
Source Data Fig. 4 Numerical source data.
Source Data Fig. 4 Unprocessed western blots.
Source Data Fig. 5 Numerical source data.
Source Data Fig. 6 Numerical source data.
Source Data Fig. 7 Numerical source data.
Source Data Extended Data Fig. 1 Numerical source data.
Source Data Extended Data Fig. 2 Numerical source data.
Source Data Extended Data Fig. 2 Unprocessed western blots.
Source Data Extended Data Fig. 3 Numerical source data.
Source Data Extended Data Fig. 4 Numerical source data.
Source Data Extended Data Fig. 5 Numerical source data.
Source Data Extended Data Fig. 5 Unprocessed western blots.
Source Data Extended Data Fig. 6 Numerical source data.

## Extended data

are available for this paper at 10.1038/s42255-022-00558-0.
