# Author Correction: The gut microbial metabolite formate exacerbates colorectal cancer progression

**DOI:** 10.1038/s42255-023-00898-5

**Published:** 2023-09-14

**Authors:** Dominik Ternes, Mina Tsenkova, Vitaly Igorevich Pozdeev, Marianne Meyers, Eric Koncina, Sura Atatri, Martine Schmitz, Jessica Karta, Maryse Schmoetten, Almut Heinken, Fabien Rodriguez, Catherine Delbrouck, Anthoula Gaigneaux, Aurelien Ginolhac, Tam Thuy Dan Nguyen, Lea Grandmougin, Audrey Frachet-Bour, Camille Martin-Gallausiaux, Maria Pacheco, Lorie Neuberger-Castillo, Paulo Miranda, Nikolaus Zuegel, Jean-Yves Ferrand, Manon Gantenbein, Thomas Sauter, Daniel Joseph Slade, Ines Thiele, Johannes Meiser, Serge Haan, Paul Wilmes, Elisabeth Letellier

**Affiliations:** 1 Molecular Disease Mechanisms Group, Department of Life Sciences and Medicine, Faculty of Science, Technology and Medicine, University of Luxembourg, Belvaux, Luxembourg; 2 School of Medicine, National University of Ireland, Galway, Ireland; 3 Ryan Institute, National University of Galway, Galway, Ireland; 4 Cancer Metabolism Group, Department of Cancer Research, Luxembourg Institute of Health, Luxembourg, Luxembourg; 5 Department of Biochemistry, Virginia Polytechnic Institute and State University, Blacksburg, VA USA; 6 Systems Ecology Group, Luxembourg Centre for Systems Biomedicine, University of Luxembourg, Esch-sur-Alzette, Luxembourg; 7 Integrated BioBank of Luxembourg, Dudelange, Luxembourg; 8 National Center of Pathology, Laboratoire National de Santé, Dudelange, Luxembourg; 9 Department of Surgery, Centre Hospitalier Emile Mayrisch, Esch-sur-Alzette, Luxembourg; 10 Clinical and Epidemiological Investigation Center, Department of Population Health, Luxembourg Institute of Health, Luxembourg, Luxembourg; 11 Discipline of Microbiology, School of Natural Sciences, National University of Ireland, Galway, Ireland; 12 APC Microbiome, Cork, Ireland

**Keywords:** Cancer metabolism, Bacterial host response, Colorectal cancer, Metabolism

Correction to: *Nature Metabolism* 10.1038/s42255-022-00558-0. Published 18 April 2022.

In the version of the article originally published, the cell line HT-29 was wrongly referred to as T18. This was identified as a result of quality control verification of the RNA sequencing datasets, where the analysis revealed the cells used were HT-29 instead of the reported T18 cells. This has now been corrected throughout the Results, Methods, Fig. 2d–f, and Extended Data figures in the HTML and PDF versions of the article. The authors confirm that this change does not alter the central findings of the manuscript.

