# Supplementary material for: The gut microbial metabolite formate exacerbates colorectal cancer progression
Source: Nat Metab. 2022 Apr 18;4(4):458–75. doi: 10.1038/s42255-022-00558-0 (PMC9046088; doi:10.1038/s42255-022-00558-0)
Supplement: Supplementary file 1 — Reporting Summary [file 42255_2022_558_MOESM1_ESM.pdf]

nature portfolio

Corresponding author(s): Elisabeth Letellier

Last updated by author(s): 11/02/2022

Reporting Summary

Nature Portfolio wishes to improve the reproducibility of the work that we publish. This form provides structure for consistency and transparency in reporting. For further information on Nature Portfolio policies, see our Editorial Policies and the Editorial Policy Checklist.

Statistics

For all statistical analyses, confirm that the following items are present in the figure legend, table legend, main text, or Methods section.

n/a	Confirmed	
☐	☒	The exact sample size (*n*) for each experimental group/condition, given as a discrete number and unit of measurement
☐	☒	A statement on whether measurements were taken from distinct samples or whether the same sample was measured repeatedly
☐	☒	The statistical test(s) used AND whether they are one- or two-sided<br>*Only common tests should be described solely by name; describe more complex techniques in the Methods section.*
☒	☐	A description of all covariates tested
☐	☒	A description of any assumptions or corrections, such as tests of normality and adjustment for multiple comparisons
☐	☒	A full description of the statistical parameters including central tendency (e.g. means) or other basic estimates (e.g. regression coefficient) AND variation (e.g. standard deviation) or associated estimates of uncertainty (e.g. confidence intervals)
☐	☒	For null hypothesis testing, the test statistic (e.g. *F*, *t*, *r*) with confidence intervals, effect sizes, degrees of freedom and *P* value noted<br>*Give P values as exact values whenever suitable.*
☒	☐	For Bayesian analysis, information on the choice of priors and Markov chain Monte Carlo settings
☒	☐	For hierarchical and complex designs, identification of the appropriate level for tests and full reporting of outcomes
☐	☒	Estimates of effect sizes (e.g. Cohen's *d*, Pearson's *r*), indicating how they were calculated

Our web collection on statistics for biologists contains articles on many of the points above.

Software and code

Policy information about availability of computer code

Data collection	GC-MS chromatograms were processed using Agilent MassHunter Quantitative Analysis for GC-MS, Version B.08.00<br>LC-MS Data were acquired with Thermo Xcalibur software (version 4.3.73.11)<br>WB: detection was performed with the Fusion FX imaging platform<br>Flow Cytometric analysis was performed using CantoII and a Fortessa FACS machine<br>Incucyte Live-Cell Analysis System (Essen Bioscience)<br>Real-Time PCR system (Applied Biosciences)<br>Sequencing of the V3 and V4 regions of prokaryotic 16S rRNA gene was performed on the Illumina MeSeq platform using 2x300 bp paired-end reads.<br>RNA sequencing: RNA library quantity and quality were assessed with the Agilent 2100 BioAnalyzer (Agilent, USA). RNA libraries were sequenced as 100bp paired-end runs on an Illumina HiSeq2500 platform.
Data analysis	Metabolite Detector software (version 3.220180913) used for mass spectrometric data post processing, quantification, MID calculations,<br>Image studio lite software for image anaylsis (WB)<br>Image J 1.53K<br>MATLAB (R2018B)<br>Cobra 3.0<br>FlowJo software 10.6.1<br>BD FACSDiva (8.0.1)<br>Raw 16S rRNA gene sequences (FASTAQ files) were cleaned and clustered as operational taxonomic units (OTUs) using NgTax or the Dada2 pipeline.<br>Cedex<br>R studio (1.2.5042, Rstudio Team 2020) with R CRAN (4.0.3, R Core Team 2020)<br>Incucyte ZOOM software (2018B, Sartorius, Germany)

RT-qPCR data was analyzed using qBase+ 3.2 (Biogazelle) according to MIQE guidelines
GraphPad Software version8
All scripts, codes and models can be found under https://gitlab.lcsb.uni.lu/mdm/ternes_et_al_2022/.

For manuscripts utilizing custom algorithms or software that are central to the research but not yet described in published literature, software must be made available to editors and reviewers. We strongly encourage code deposition in a community repository (e.g. GitHub). See the Nature Portfolio guidelines for submitting code & software for further information.

Data

Policy information about availability of data

All manuscripts must include a data availability statement. This statement should provide the following information, where applicable:

- Accession codes, unique identifiers, or web links for publicly available datasets
- A description of any restrictions on data availability
- For clinical datasets or third party data, please ensure that the statement adheres to our policy

All data that support the findings of this study are available in the source data provided with the manuscript. Additionally, sequence data from the EGA is available under accession number EGAS00001000288. Sequence data from the CRC patients and healthy donor samples can be found on ENA (EBI) under the accession number PRJEB46665. The TCGA analysis was based upon data (TCGA-COAD, TCGA-READ) generated the TCGA Research Network: https://www.cancer.gov/tcga. All scripts and models can be found under https://gitlab.lcsb.uni.lu/mdm/ternes_et_al_2022/. Microbial reconstructions are modified from the virtual Metabolic Human website (www.vmh.life) and available on gitlab.

Field-specific reporting

Please select the one below that is the best fit for your research. If you are not sure, read the appropriate sections before making your selection.

☒ Life sciences ☐ Behavioural & social sciences ☐ Ecological, evolutionary & environmental sciences

For a reference copy of the document with all sections, see nature.com/documents/nr-reporting-summary-flat.pdf

Life sciences study design

All studies must disclose on these points even when the disclosure is negative.

Sample size	For in vitro studies a minimum of triplicates per condition were used and each experiment was verified in at least three independent experiments. For in vivo work, the number of mice per group was calculated based on a power calculation in liaison with a bio-statistician. Please refer to the Figure legends and Method sections for further details.
Data exclusions	no data was excluded unless clear technical issues were identified
Replication	All in vitro experiments were verified in at least three independent experiments with same overall outcome. Specific number of repetitions is provided in the manuscript. For in vivo groups, adequate group size was calculated beforehand. As stated in the manuscript, for some experiments, we pooled two independent experiments to reach the number calculated by the bioinformatician. All in vivo replication were successful.
Randomization	Before starting all of the in vivo experiments, mice were randomly allocated to different groups. All Mass Spec samples were randomized during the analysis to avoid bias from eg. instrument drift.
Blinding	Analyses of in vivo samples (tumor counts) were blinded and analyzed by two researchers independently. IDs were uncovered after final result was obtained. Results from both persons lead to the same overall result.

Reporting for specific materials, systems and methods

We require information from authors about some types of materials, experimental systems and methods used in many studies. Here, indicate whether each material, system or method listed is relevant to your study. If you are not sure if a list item applies to your research, read the appropriate section before selecting a response.

Materials & experimental systems

n/a	Involved in the study	
☐	☒	Antibodies
☐	☒	Eukaryotic cell lines
☒	☐	Palaeontology and archaeology
☐	☒	Animals and other organisms
☐	☒	Human research participants
☒	☐	Clinical data
☒	☐	Dual use research of concern

Methods

n/a	Involved in the study	
☒	☐	ChIP-seq
☐	☒	Flow cytometry
☒	☐	MRI-based neuroimaging

Antibodies

Antibodies used	Actin,OCT4,Tubulin,ALDH1A1,ALDH2,ERK,pERK1/2,MEK,pMEK,p38,pp38,p65,pp65,CD8a,CD4,CD3e,NKp46,TNFα,CD127,IL-17a,IFNγ,CD4,CD16/32,CD25,IL-4,CD19,CD86,CD11c,CD80,CD40,IL-22,IL-2,TER119,CD11b,Tbet,Gata3,MHC II,CD11b,CD86,CD19,CD5,Ly6G,RORγT,FoxP3,F4/80, Ahr, donkey anti-rabbit Alexa Fluor 647. For further information, please refer to Supplementary Table 5.
Validation	All used antibodies are well described and used across research laboratories as well as used in published manuscripts.

Eukaryotic cell lines

Policy information about cell lines

Cell line source(s)	The HT-29, HCT116 and CACO-2commercial CRC cell lines were obtained from ATCC and maintained under the in DMEMF12 with 10% [v/v] FBS and 1% [v/v] penicillin/streptomycin. RKO-7TGP were received from the University of Vienna (Dolznig lab) and maintained as described in the manuscript.
Authentication	All cell lines except the primary cultures established have been received from ATCC and authenticated via STR profiling.
Mycoplasma contamination	Cells have been checked for mycoplasma every two weeks and all cell lines used in the manuscript were mycoplasma negative.
Commonly misidentified lines (See ICLAC register)	HCT116 are part of the list but have been authenticated via STR profiling at DSMZ before the study.

Animals and other organisms

Policy information about studies involving animals; ARRIVE guidelines recommended for reporting animal research

Laboratory animals	mouse musculus C57Bl6N and NSG mice both male and female aged 11-15 and 7-8 respectively.
Wild animals	No wild animals were used
Field-collected samples	No field collected samples were used
Ethics oversight	All animal experiments were performed according to all applicable laws and regulations, after receiving approval by the institution's Animal Experimentation Ethics Committee at UL (AEEC) and the Ministry of Agriculture, Viniculture & Rural Development (LUPA 2019/99 and LUPA 2019/60). They ensure that care and use of animals for research purposes was conducted according to the EU Directive 2010/63/EU, as well as the Grand-Ducal Regulation of January 11, 2013 on the protection of animals used for scientific purposes. These included the justification of the use of animals, their welfare and the incorporation of the principles of the 3R's (Replacement, Reduction and Refinement). A biostatistician reviewed all animal protocols.

Note that full information on the approval of the study protocol must also be provided in the manuscript.

Human research participants

Policy information about studies involving human research participants

Population characteristics	The average ages of stool sample donors were 70.29 +/- 8.74, with 12 female and 40 male subjects (in-house CRC collection) and 62.05 +/- 7.51, with 30 female and 33 male subjects (ND collection). All patients were enrolled between the years 2010 and 2019.
Recruitment	All CRC patients were enrolled at diagnosis by the Centre Hospitalier Emile Mayrisch (Esch-sur-Alzette, Luxembourg), the Centre Hospitalier de Luxembourg (Luxembourg, Luxembourg), or the Zitah Klinic (Luxembourg, Luxembourg). A healthy control cohort (ND collection) was enrolled by the National Centre of Excellence in Research on Parkinson's disease (NCER-PD) and 16S rRNA gene sequencing data was shared according to a biomaterial cooperation agreement.
Ethics oversight	Patient samples were donated willingly under informed consent and were handled in accordance with institutional guidelines. Ethical approval was obtained from the Comité National d'Ethique de Recherche, Luxembourg (Reference 201009/09) followed by institutional approval by the Ethics Review Panel of the University of Luxembourg (ERP-16-032).

Note that full information on the approval of the study protocol must also be provided in the manuscript.

Flow Cytometry

Plots

Confirm that:

☒ The axis labels state the marker and fluorochrome used (e.g. CD4-FITC).

☒ The axis scales are clearly visible. Include numbers along axes only for bottom left plot of group (a 'group' is an analysis of identical markers).

☒ All plots are contour plots with outliers or pseudocolor plots.

☒ A numerical value for number of cells or percentage (with statistics) is provided.

Methodology

Sample preparation	Organoids embedded in Matrigel were treated with 5mM sodium formate (Carl Roth, 4404.1), for 48h at 37°C. ALDH activity was assessed according to the manufacturer's protocol (ALDEFLUOR Kit, Stemcell Technologies, 01700). CD133 (Miltenyi Biotec, 130-090-826), CD44 (BD Biosciences, 555428) and CD24 (BD Biosciences, 560533) labeling was done at a 1:50 dilution. LIVE/DEAD cell viability staining was performed at a 1:1000 dilution (Invitrogen, L34975). Samples were acquired on a FACS CantoII Cell Analyzer (BD Biosciences). Results were analyzed with the FlowJo software (BD Biosciences).<br><br>Xenografts were harvested at endpoint and dissociated (Human Tumor Dissociation Kit, Miltenyi) for further FACS-based protein expression analysis.<br><br>Colons were opened longitudinally, washed with cold PBS, cut into smaller sections and digested first in RPMI containing glutamine/GlutamMAX, 3% FBS, 1% P/S, 5mM EDTA and 0.154mg/mL DTT, then in serum-free RPMI with Liberase (0.1mg/mL) and DNAse (0.5mg/mL), both in a shaking incubator at 800rpm and 37°C for 20min and 30min respectively. Spleens were digested in serum-free RPMI with Liberase (0.1mg/mL) and DNAse (0.5mg/mL) for 30min. Digestions were quenched with RPMI medium containing 10% FBS and cell suspensions were filtered through a 70µm filter. Mesenteric lymph nodes were mashed through a 70µm filter. Single-cell suspensions were restimulated in RPMI containing 10% FBS, 0.1µg/mL PMA and 1.5µg/mL ionomycin for 4h at 37°C. Brefeldin A was added 1h after the beginning of the restimulation. Cells were stained with Near IR fixable live/dead dye and with the corresponding antibody panels (Supplementary Table 6) using the Cytofix/Cytoperm kit (554714, BD Bioscience). An additional Fc blocking step preceded antibody staining for macrophages and dendritic cells. Acquisition was performed on a CantoII and a Fortessa FACS machine and data was analyzed using BD FACSDiva 8.0.1 and FlowJo software 10.6.1 (gating strategies can be found in Extended Data Figures 7 and 8).
Instrument	BD FACS CantoII and BD LSR Fortessa FACS machine
Software	BD FACS Diva 8.0.1 and FlowJo 10.6.1 softwares
Cell population abundance	Total cell counts were determined by Cedex and multiplied by frequency of subpopulations to get absolute counts.
Gating strategy	FSC/SSC selection of cells, exclusion of doublets by FSC-A/FSC-H<br>Unstained controls and single stained controls were acquired to identify negative and positive cell populations for each marker.<br>Detailed gating strategy is explained in the extended data figure 7-8

☒ Tick this box to confirm that a figure exemplifying the gating strategy is provided in the Supplementary Information.
