# Supplementary figures and images for: The gut microbial metabolite formate exacerbates colorectal cancer progression
Source: Nat Metab. 2022 Apr 18;4(4):458–75. doi: 10.1038/s42255-022-00558-0 (PMC9046088; doi:10.1038/s42255-022-00558-0)

ALDH1A1

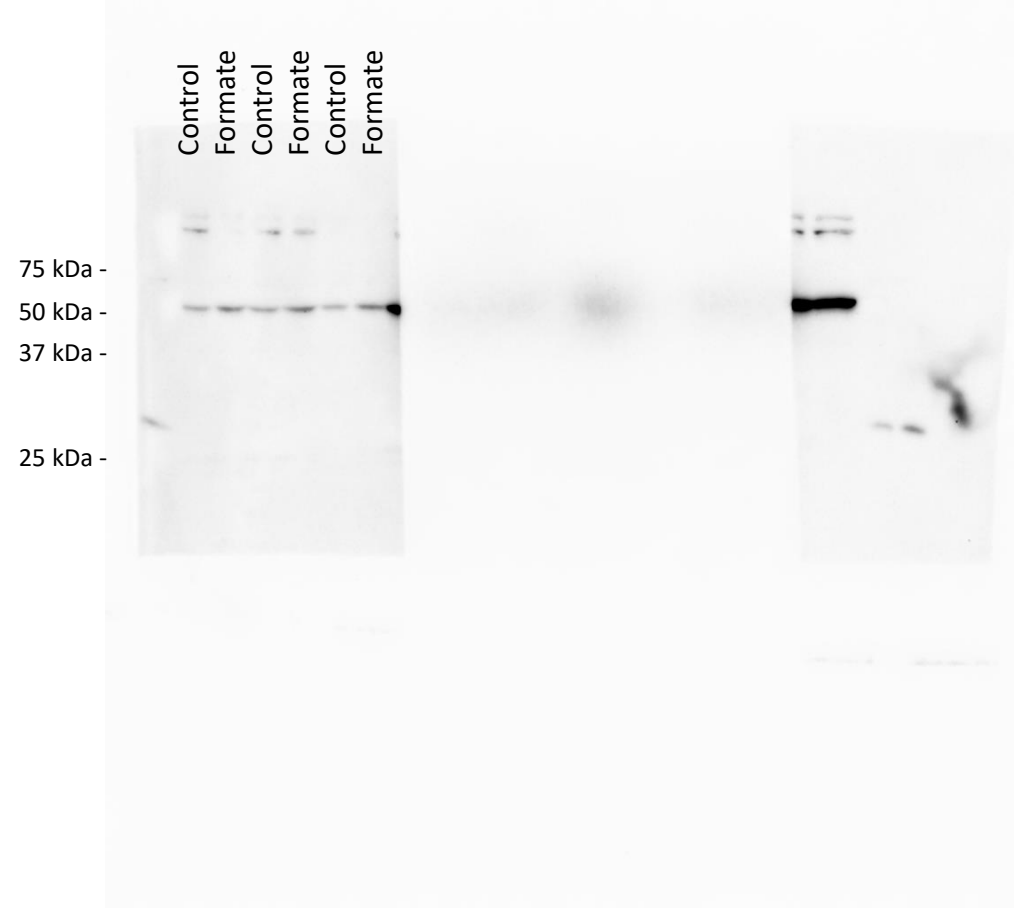

Actin

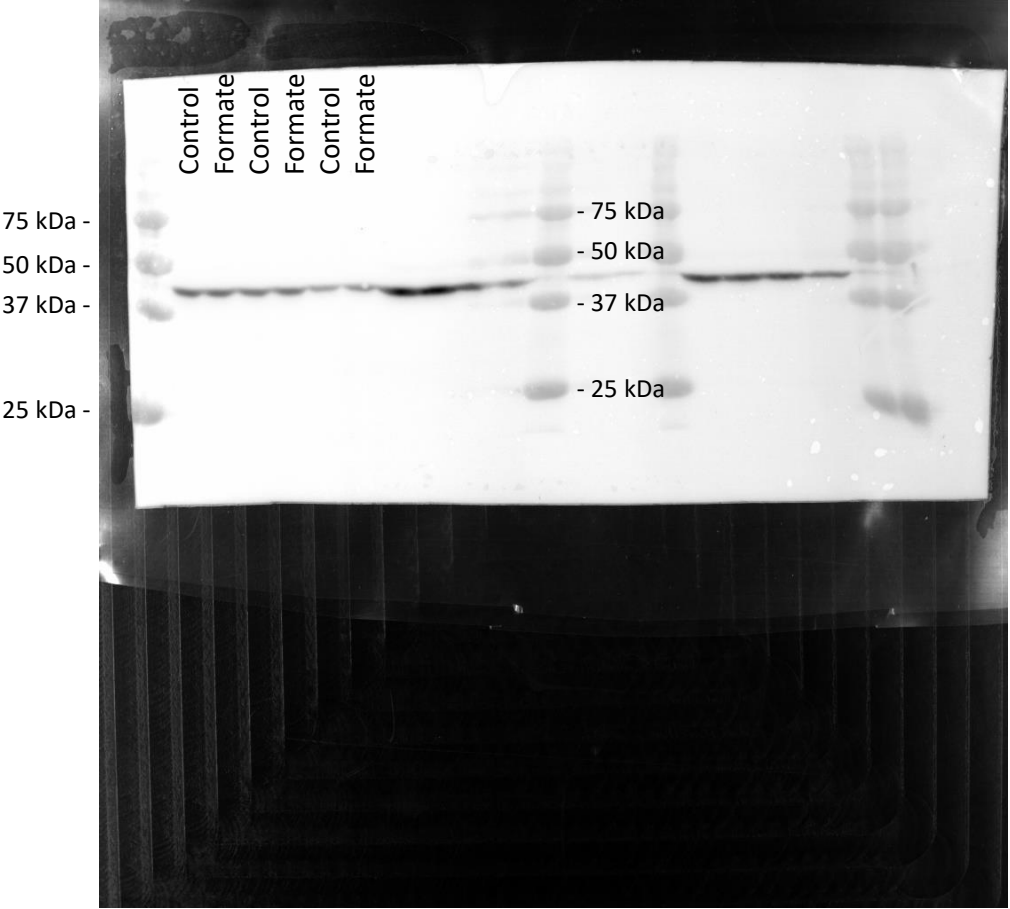

Supplement: Source Data Fig. 4 — Unprocessed western blots. [file 42255_2022_558_MOESM7_ESM.pdf]

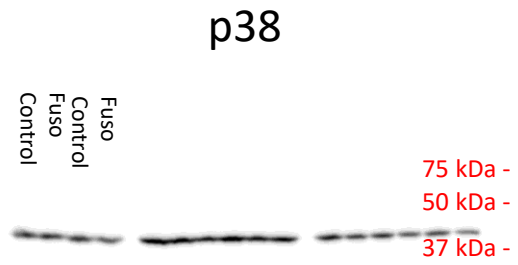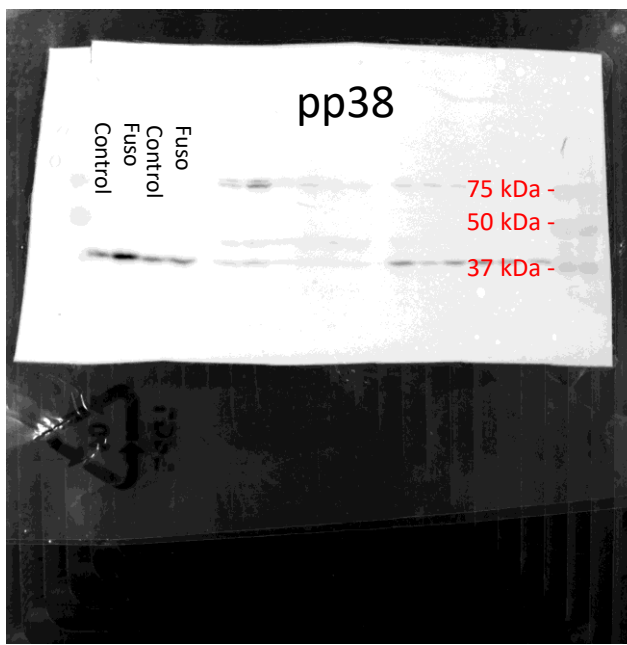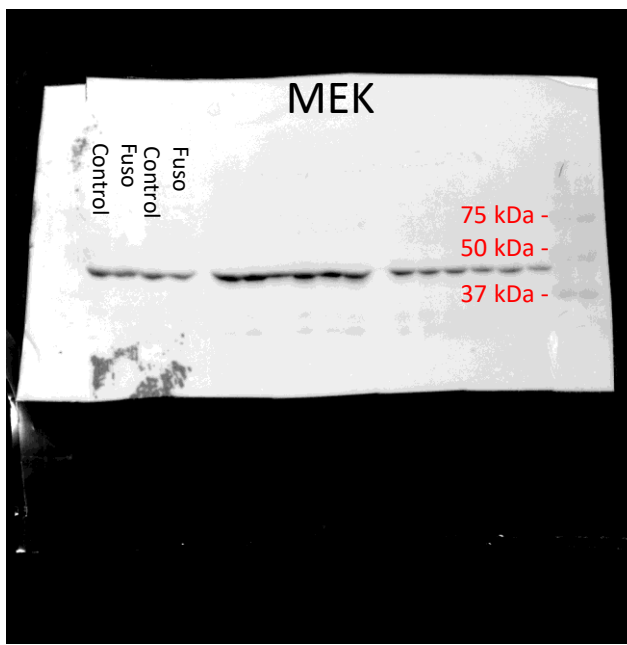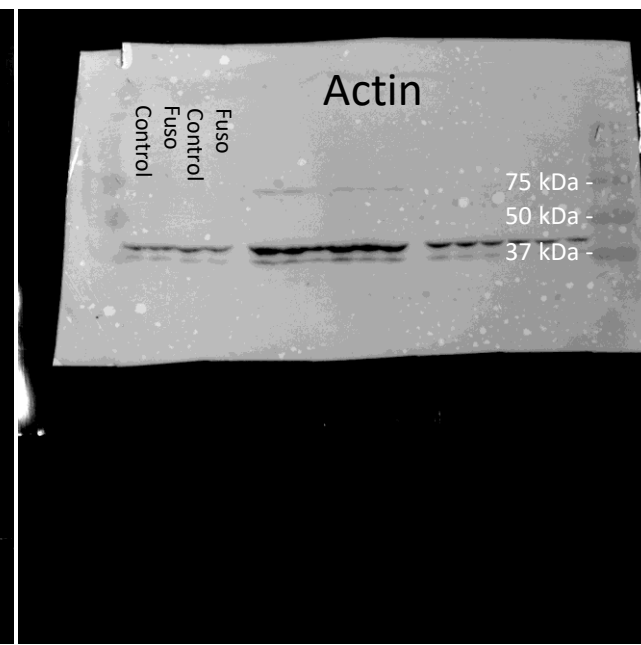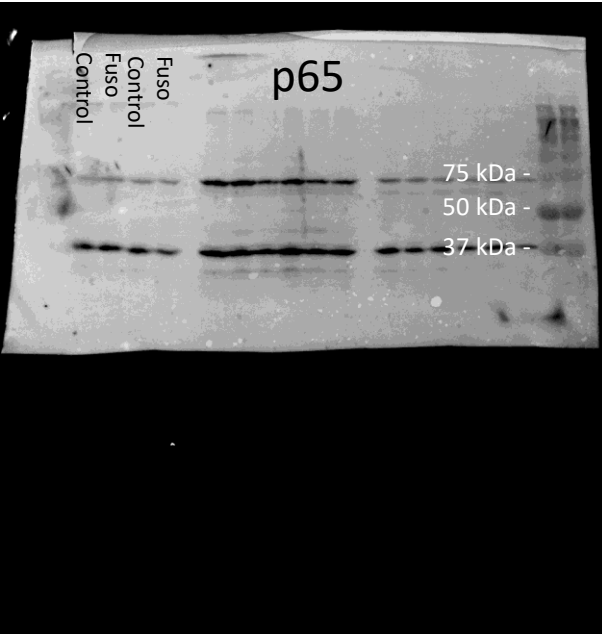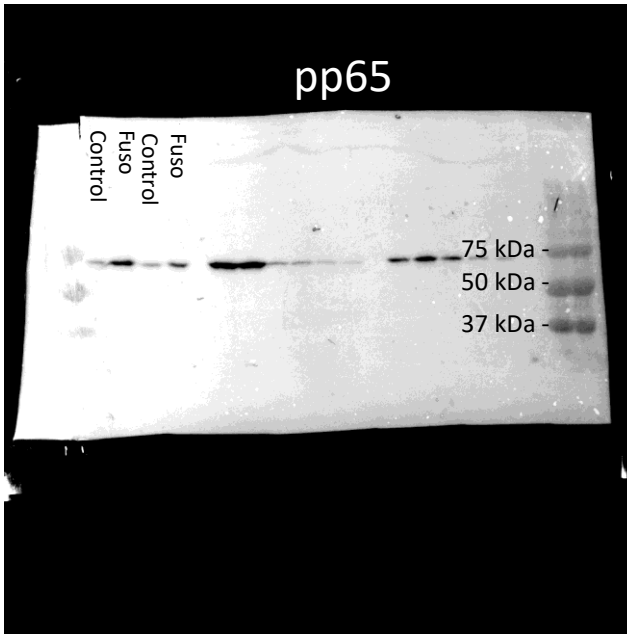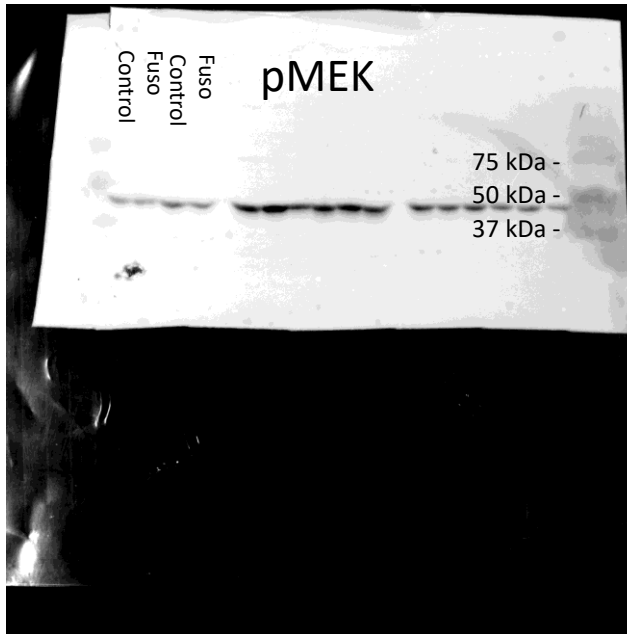

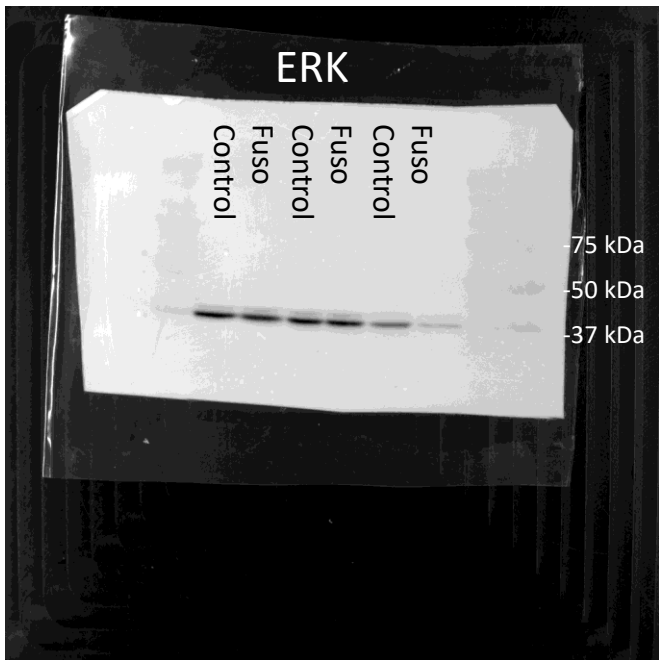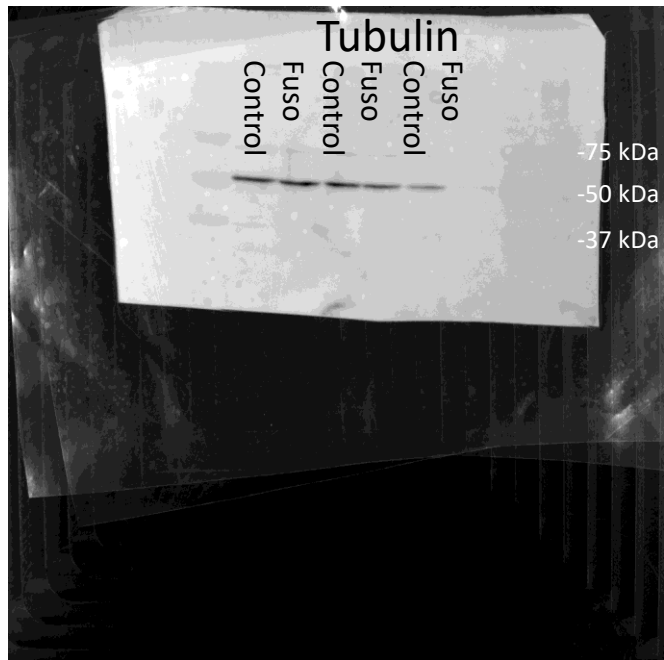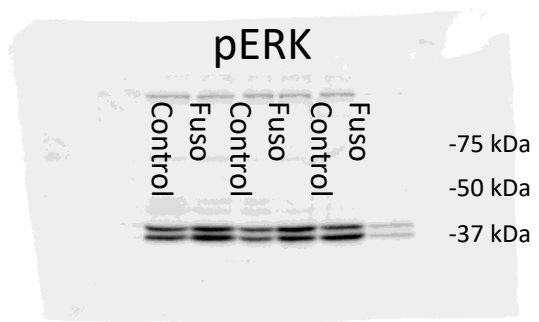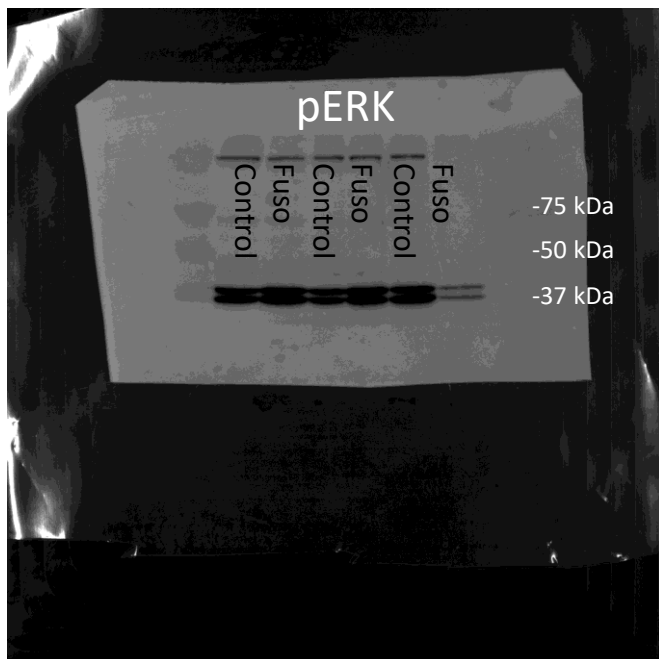

Supplement: Source Data Extended Data Fig. 2 — Unprocessed western blots. [file 42255_2022_558_MOESM13_ESM.pdf]

Actin

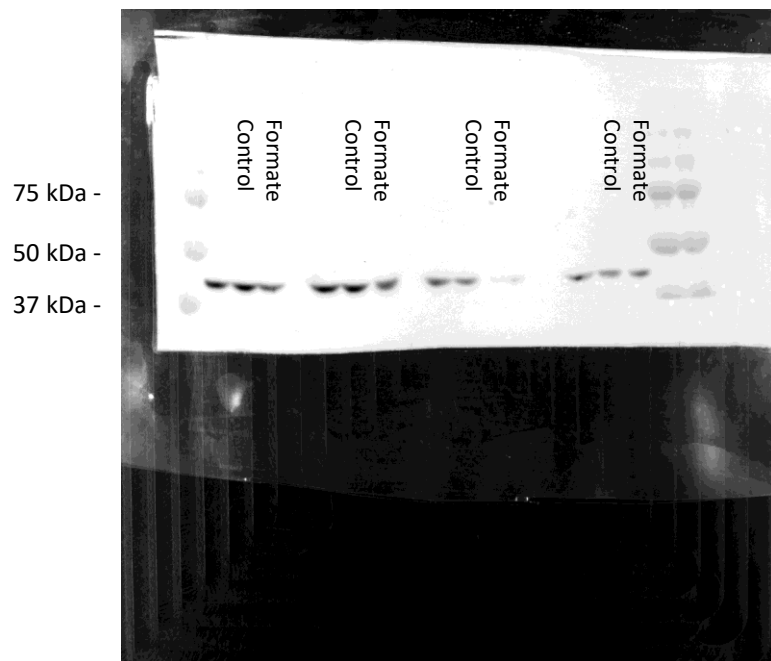

ALDH2

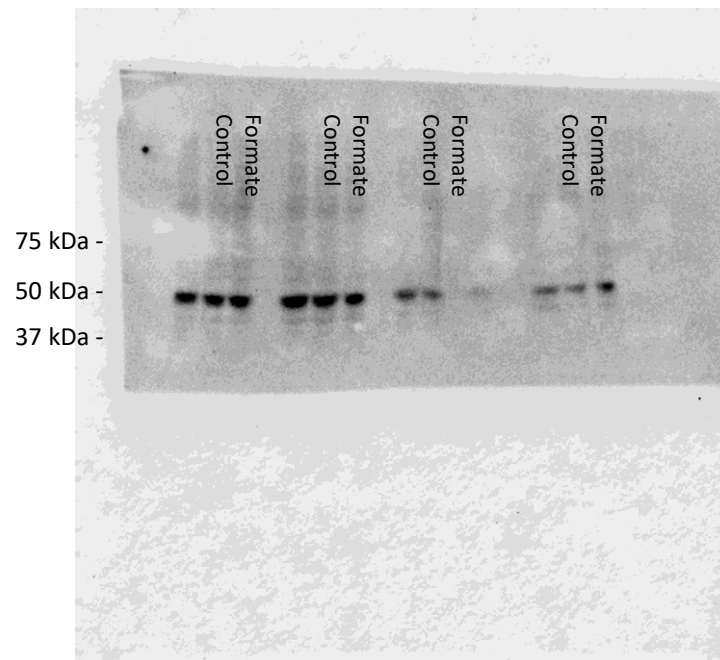

ALDH2

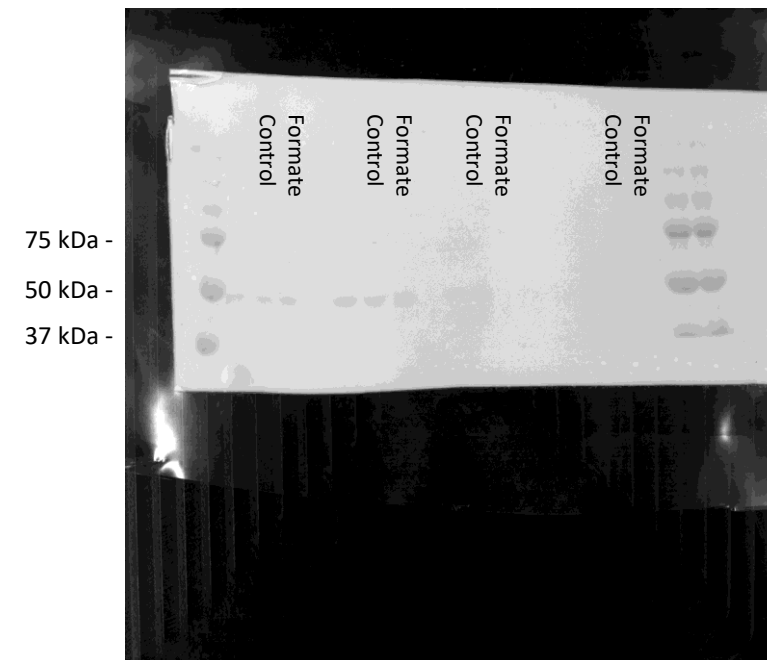

Actin

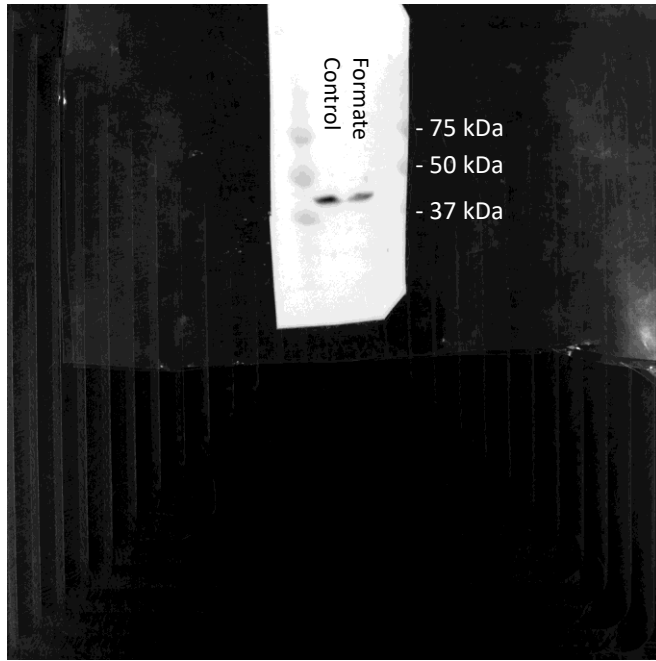

ERK

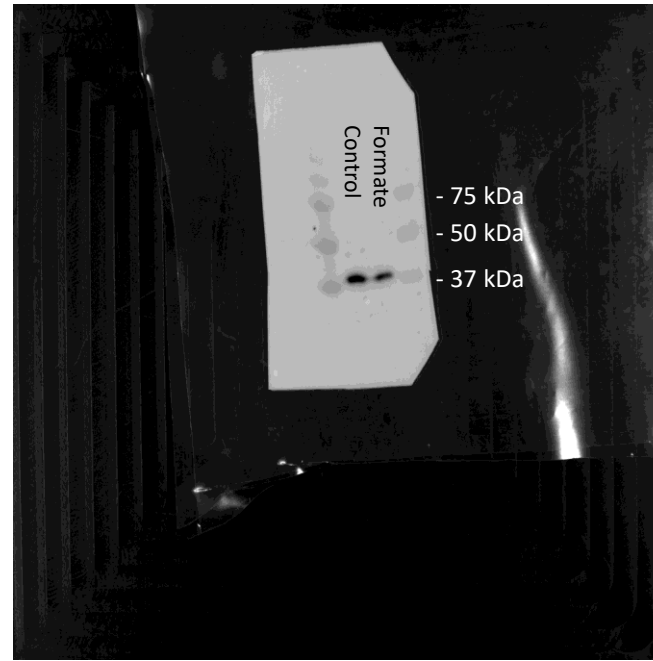

pERK

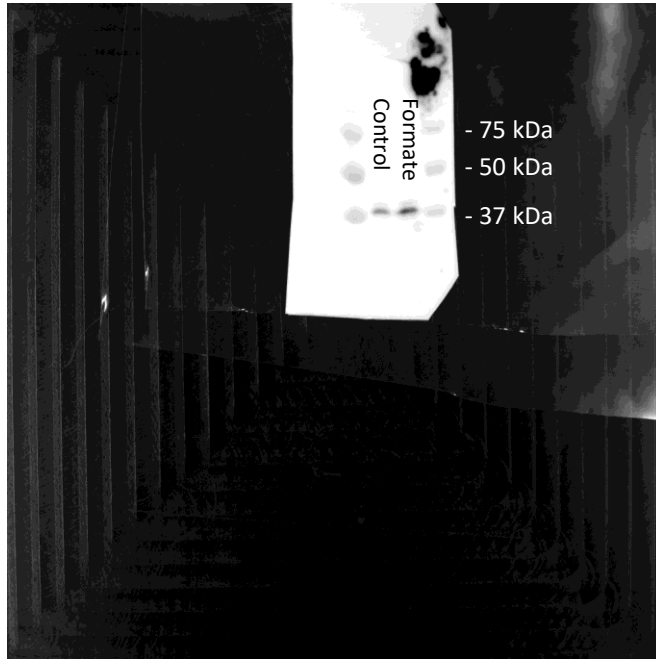

pERK

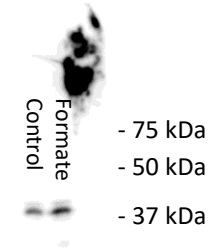

Actin

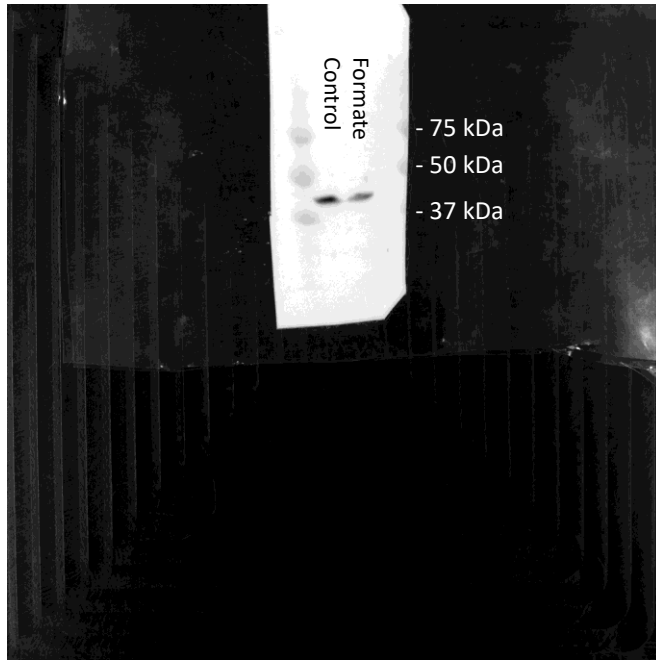

ERK

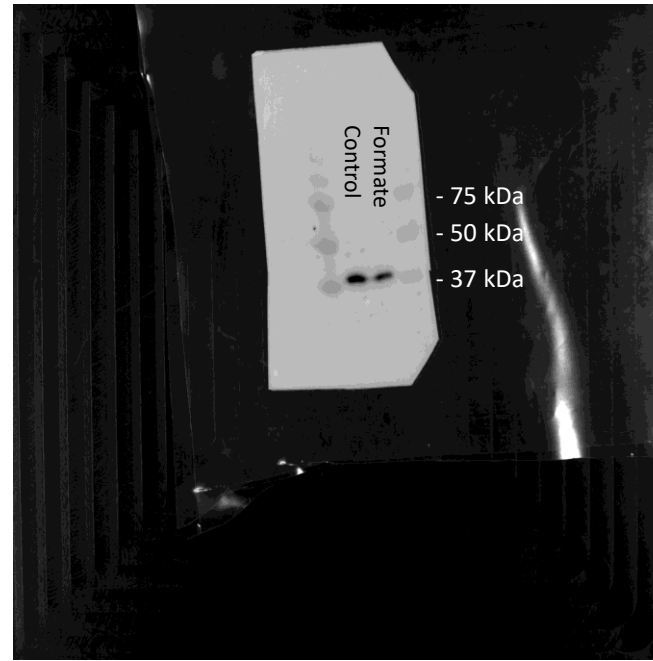

pERK

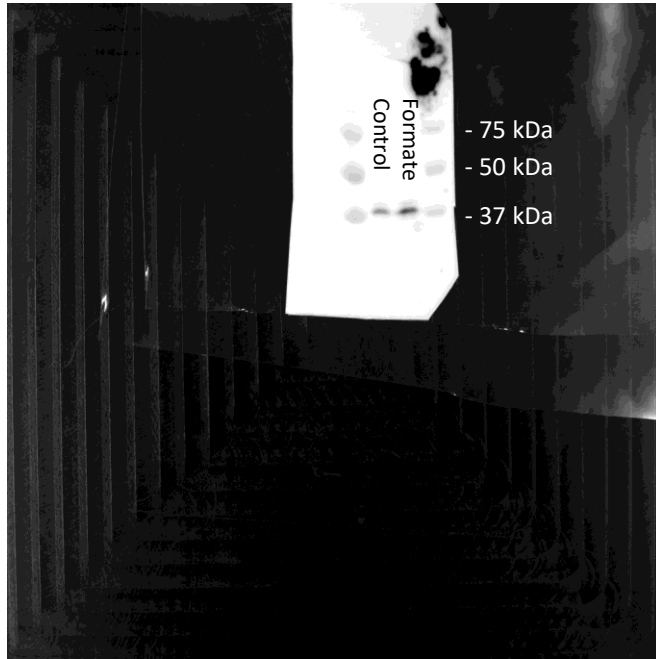

pERK

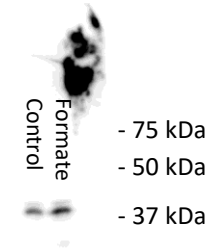

Actin

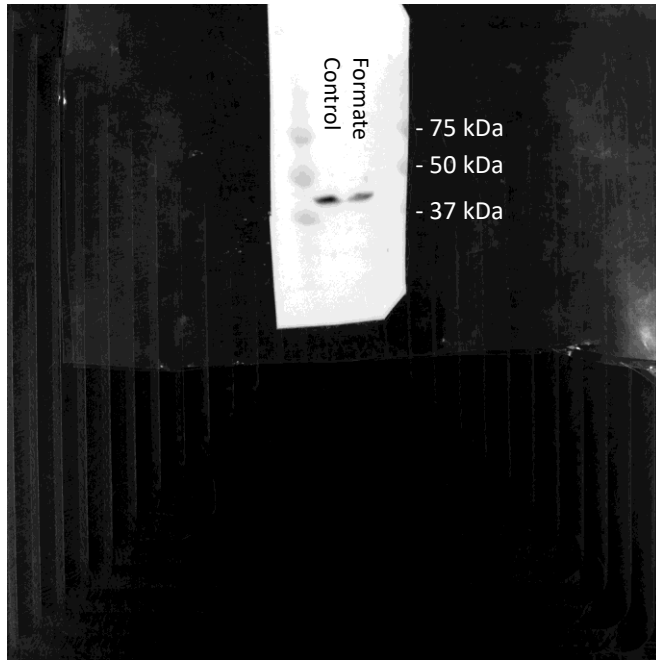

ERK

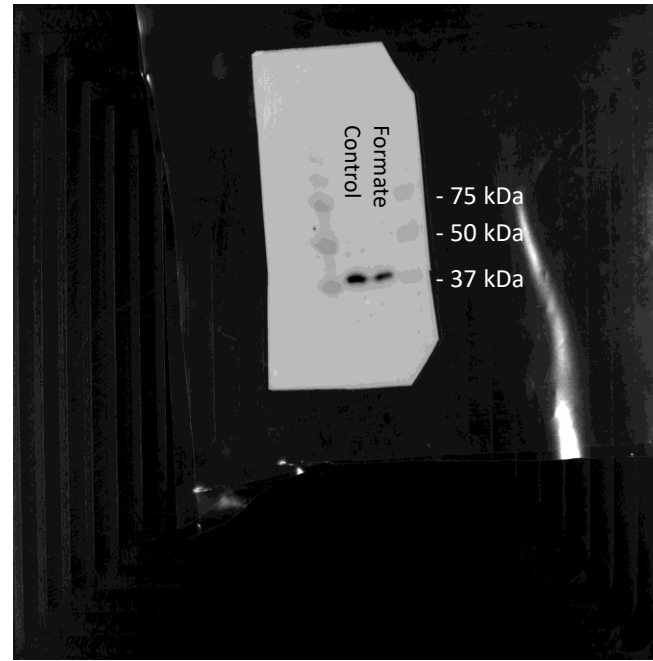

pERK

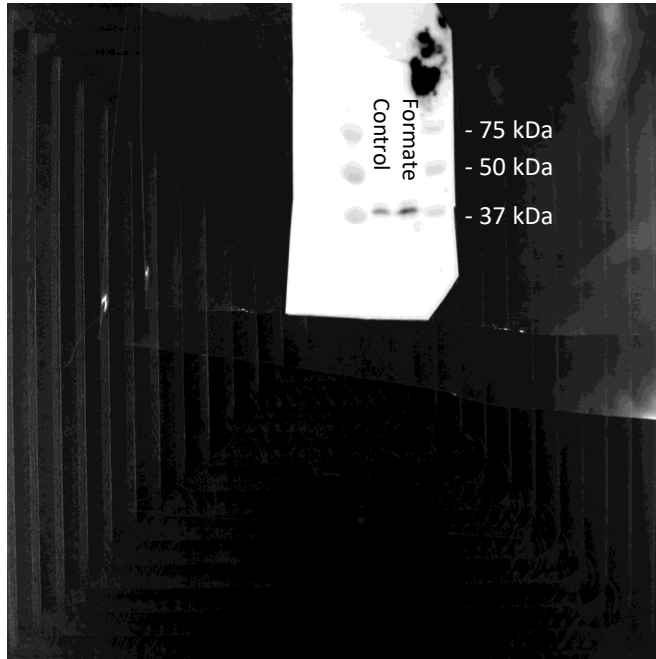

pERK

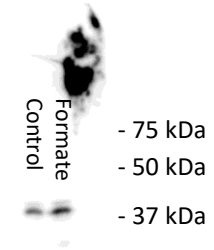

Supplement: Source Data Extended Data Fig. 5 — Unprocessed western blots. [file 42255_2022_558_MOESM17_ESM.pdf]
